# HBV-driven host chromatin accessibility changes affect liver metabolic pathways, iron homeostasis and promote a preneoplastic phenotype

**DOI:** 10.1186/s13046-025-03414-7

**Published:** 2025-05-16

**Authors:** Vincenzo Alfano, Giuseppe Rubens Pascucci, Giacomo Corleone, Massimiliano Cocca, Francesca De Nicola, Océane Floriot, Alexia Paturel, Francesca Casuscelli Di Tocco, Claude Caron de Fromentel, Philippe Merle, Michel Rivoire, Massimo Levrero, Francesca Guerrieri

**Affiliations:** 1 IHU EVEREST - Institut of Hepatology Lyon, UMR UCLB1 INSERM U1350 PaThLiv, 69004 Lyon, France; 2 Present Address: INSERM U1052, Cancer Research Center of Lyon (CRCL), Lyon, France; 3 Research Unit of Clinical Immunology and Vaccinology, Bambino Gesù Children’s Hospital, 00165 Rome, Italy; 4 Center for Life Nano Science (CNLS), Istituto Italiano Di Tecnologia (IIT), 00161 Rome, Italy; 5 SAFU Laboratory, Department of Research, Advanced Diagnostics, and Technological Innovation, IRCCS Regina Elena National Cancer Institute, 00144 Rome, Italy; 6 Université Catholique de Lyon (UCLy), 69002 Lyon, France; 7 Department of Hepatology, Croix Rousse Hospital, Hospices Civils de Lyon, 69004 Lyon, France; 8 INSERM U1052, Centre de Lutte Contre Le Cancer Léon Bérard (CLB), 69003 Lyon, France

**Keywords:** Chromatin remodeling, ATAC-seq, RNA-seq, Iron uptake, HBV-related HCC

## Abstract

**Backround and aims:**

Complex host-virus interactions account for adaptive and innate immunity dysfunctions and viral cccDNA mini-chromosome persistence, key features of HBV chronicity and challenges for HBV cure. The extent of HBV direct impact on liver transcriptome remains controversial. Transcriptional activation in eukaryotic cells is tightly linked with disruption of nucleosome organization at accessible genomic sites of remodeled chromatin. We sought to investigate the impact of HBV on chromatin accessibility and transcription.

**Methods:**

We used ATAC-seq (Assay for Transposase Accessible Chromatin followed by high throughput sequencing) to detect early changes in chromatin accessibility coupled with RNA-seq in HBV-infected Primary Human Hepatocytes (PHHs).

**Results:**

An increasing number of genomic sites change their nucleosome organization over time after HBV infection, with a prevalent, but not exclusive, reduction of chromatin accessibility at specific sites that is partially prevented by inhibiting HBV transcription and replication. ATAC-seq and RNA-seq integration showed that HBV infection impacts on liver fatty acids, bile acids, iron metabolism and liver cancer pathways. The upregulation of iron uptake genes leads to a significant increase of iron content in HBV-infected PHHs whereas iron chelation inhibits cccDNA transcription and viral replication. The chromatin accessibility and transcriptional changes imposed by HBV early after infection persist, as an epigenetic scar, in chronic HBV (CHB) patients and in HBV-related HCCs. These changes are to a large extent independent from viral replication levels and disease activity.

**Conclusions:**

Altogether our results show that HBV infection impacts on host cell chromatin landscape and specific transcriptional programs including liver metabolism and liver cancer pathways. Re-wiring of iron metabolism boosts viral replication early after infection. The modulation of genes involved in cancer-related pathways may favor the development or the selection of a pro-neoplastic phenotype and persists in HBV-related HCCs.

**Supplementary Information:**

The online version contains supplementary material available at 10.1186/s13046-025-03414-7.

## Introduction

Close to 300 million individuals have chronic hepatitis B (CHB) worldwide and the World Health Organization (WHO) estimates up to one million deaths per year by 2030 from HBV-associated liver diseases, such as cirrhosis and hepatocellular carcinoma (HCC) [1]. HBV vaccination is safe and effective, but coverage is still limited in many countries. Nucleos(t)ide analogs (NUCs), efficiently inhibit the viral reverse transcriptase and impact on disease progression but do not achieve a complete clearance of the virus and require long term/lifelong treatment to avoid viral reactivations [2]. The nuclear covalently closed circular DNA (cccDNA) replicative intermediate, which is the template for transcription of viral RNAs for protein production and generation of new viral genomes, is not directly affected by NUCs [3]. New antiviral and immuno-modulatory compounds aiming at functional or complete HBV cure are in clinical development [4, 5]. HBV-specific T and B cell dysfunctions and the failure to achieve a complete elimination of the infected hepatocytes carrying the cccDNA are responsible for HBV chronicization and represent the major obstacles for HBV cure [3–5].

Lack or low sensing of HBV by the innate immune system and HBV ability to inhibit signaling through pattern recognition receptor (PRRs) pathways likely contribute to deficient immune responses in CHB [6]. The limited or no induction of the innate immune response following acute HBV infection in chimpanzees [7], in chimeric mice carrying a humanized liver [8], in woodchucks acutely infected with the woodchuck hepatitis virus (WHV) [9] as well as in HBV infected primary human hepatocytes in vitro [10] and in fresh liver biopsies ex vivo [11] has led to the widely accepted concept that HBV is a"stealth"virus that induces no or very few changes in infected cells [12]. Despite some studies have shown that HBV is indeed detected by innate immunity sensors [13, 14] the notion that HBV does not induce a strong innate immune response and does not activate class I interferon signaling holds. Conversely, HBV has been consistently shown to have an impact on host cells transcription in infection studies in vitro [15–17]. Whereas liver transcriptomic changes in acute HBV hepatitis have not been investigated due to the obvious limitations in the access to patients’ samples, in untreated CHB patients there is a strong repression of innate immune response genes [18] and the activation, in 1/3 of the cases, of class II interferons as well as the IL6/STAT3, TNFa/NFkB inflammatory pathways [19]. Although these studies confirm the presence of transcriptomic changes in the liver of CHB patients, they were obtained in a chronic situation with intrahepatic inflammation and ongoing viral replication for years, making difficult if not impossible to identify the direct contribution of HBV.

Transcriptional activation in eukaryotic cells is tightly linked with disruption of nucleosome organization at promoters, enhancers, silencers, insulators, and locus control regions. Regulatory DNA tends to coincide with open or accessible genomic sites of remodeled chromatin. Genomic chromatin accessibility assays, such as DNase-seq [20], FAIRE-seq [21], MNase-seq [22] and ATAC-seq, are based upon genome fragmentation by enzymatic or chemical means followed by the isolation of either accessible or protected loci [23, 24] and quantification using a next-generation sequencing (NGS) platform. These assays allow to identify chromatin changes associated to differential gene expression, functional diversification, and disease development [25–28]. ATAC-seq (assay for transposase-accessible chromatin followed by high throughput sequencing), the most current method for probing changes in the accessibility of native chromatin, is based on the ability of hyperactive Tn5 transposase to fragment DNA and integrate into active regulatory regions in vivo [23, 29, 30] and does not involve any chemical modification. ATAC-seq allows to study, simultaneously and at high resolution, multiples aspects of chromatin architecture and remodeling, including the detection of open chromatin and TF fingerprints on the whole genome using a few cells.

Here we applied ATAC-seq to detect early changes in host chromatin accessibility in HBV-infected primary human hepatocytes (PHHs) to identify a common HBV footprint. We show that an increasing number of genomic sites change their chromatin accessibility over time in HBV-infected cell. The prevalent chromatin remodeling effect imposed by HBV infection is a reduction of chromatin accessibility at specific sites that is partially prevented by inhibiting HBV transcription and replication. The integration of ATAC-seq and RNA-seq shows that HBV infection affects liver metabolic, liver injury and liver cancer pathways. These early changes persist in the liver of CHB patients. We also identified a 39-genes liver-iron signature modulated by chromatin accessibility changes and the upregulation of iron uptake genes. HBV infection of PHHs is accompanied by a significant increase of free iron content, and we showed that intracellular iron chelation results in a drastic inhibition of cccDNA transcription and viral replication.

## Methods

Primary culture of human hepatocytes, HBV infection and pharmacological treatments are detailed in the online Supplementary information. Chromatin Tagmentation and Sequencing, ATAC-seq data processing, RNA-seq and all bioinformatic analysis are detailed in the online Supplementary Information and graphically summarized in Figure S1. Primer pairs and probes used throughout the manuscript are listed in Table S1.

## Results

### Progressive changes in chromatin accessibility following HBV infection

**Fig. 1:**
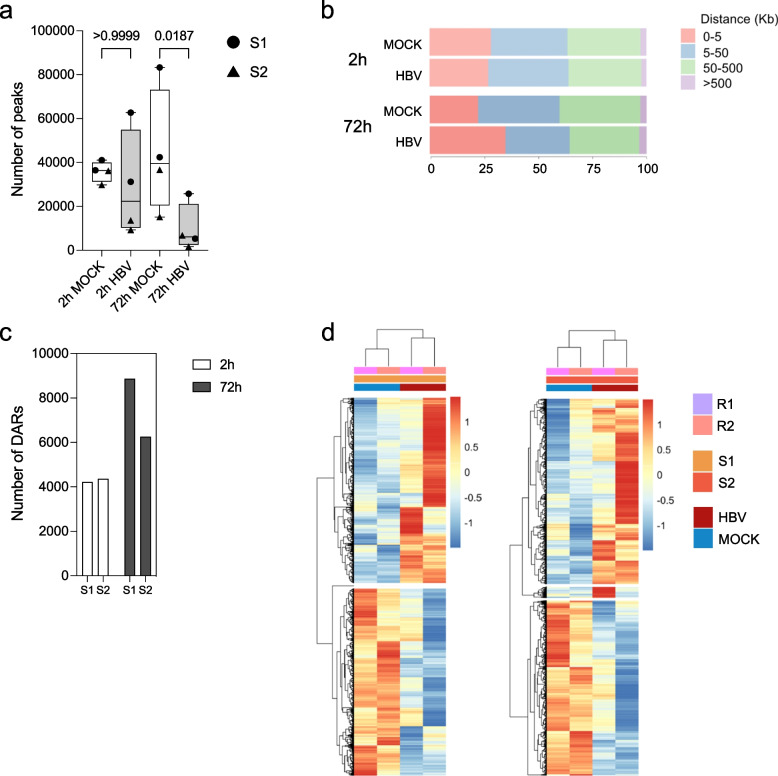
ATAC-seq analysis of chromatin accessibility changes in HBV-infected PHHs. **a** ATAC-seq peaks detected at 2 h and 72 h post infection (p.i.) in MOCK (white Box Plots) and HBV-infected (grey Box Plots) primary human hepatocytes (PHHs). Dots represent the total number of peaks detected in each replicate from donor 1 (S1). Triangles represent the total number of peaks detected in each replicate from donor 2 (S2). Sequencing reads alignment to the reference human genome hg19 and peaks calling are detailed in Supplementary Information. **b** Cumulative percentage of ATAC-seq peaks in MOCK and HBV-infected cells at 2 h and 72 h p.i. as a function of their absolute genomic distance in kilobases (kb) to the closest transcription starting site (TSS). Sequencing reads alignment to the reference human genome hg19 and peaks calling are detailed in Supplementary Information. **c**. Number of Differential Accessible Regions (DARs) (see the Supplementary Information for details) identified in HBV-infected PHHs from donor 1 (S1) and donor 2 (S2) at 2 h (*n* = 4129; 4364) and 72 h p.i. (*n* = 8873; 6256). **d** Heatmaps of CPM z-scores (see the Supplementary Information for details) of the DARs identified at 72 h in the R1 and R2 replicates from S1 (left panel) and S2 (right panel) donors in HBV (red) and MOCK (blue) conditions. CPM z-scores values are represented with a color scale from blue (negative) to red (positive)

The extent of HBV direct transcriptional reprogramming remains controversial. To gain further insights into the host nuclear response to HBV infection, we investigated the genome-wide landscape of chromatin accessibility in a relevant HBV infection model. HBV infection of primary human hepatocytes (PHHs) results in a robust viral parameters expression (Figure S2a-b). The range of infected cells, as determined by the percentage of HBc positive cells at 72 h post-infection (p.i.), ranged in the different replicates from 46% to 70.2% for sample 1 (S1) (mean 55.8% median 54.3%) and 43.3 to 79.3% for sample 2 (S2) (mean 66.2, median 71.6%), respectively. The number of ATAC-seq peaks, that denote the Chromatin Accessible Regions (CARs), detected using a standard peak calling method (see Supplemenrtary information) is markedly reduced in HBV-infected PHHs 72 h p.i. (productive infection with active 3.5 kb RNA species transcription from cccDNA and viral particles production [10]) (average ~ 9800 CARs) as compared to MOCK controls (average ~ 44,000 CARs) (Fig. 1a), despite the comparable number of total sequenced reads in the different samples (Table S2). The average number of peaks is also lower in HBV as compared to MOCK samples at 2 h p.i. (early entry and post-entry phase) [10], but the values are more dispersed (Fig. 1a).

The analysis of the detected ATAC-seq peaks as a function of their distance from the closest transcription starting site (TSS) showed a relative increase of the frequency of CARs in the proximity of TSSs (0–5 kb) at 72 h p.i. (Fig. 1b). This latter observation, coupled with the significant reduction in the number of total CARs at 72 h showed in Fig. 1a, suggests a loss of accessibility variegation at more distal loci. Principal Components Analysis (PCA) of ATAC-seq chromatin accessibility profiles (Figure S3a) shows that: i) each donor exhibits a unique repertoire of ATAC-seq peaks (compare dots, S1 vs triangles, S2); ii) the impact of HBV infection at 2 h is relatively low (compare light blue dots and triangles, corresponding to MOCK S1 and S2, vs pink dots and triangles, corresponding to HBV S1 and S2) and much more evident at 72 h p.i., when HBV samples from S1 and S2 (magenta dots and triangles) separate from both MOCK samples at 72 h p.i. (darker blue dots and triangles) and the HBV or MOCK samples at 2 h p.i..

To better exploit the information associated to each *significant* peak detected and identify the peaks that are *down-regulated* after HBV infection (i.e., their size is significantly reduced, or they are not anymore called at all) or *up-regulated* after HBV infection (i.e., their size is significantly increased, or they are newly detected and called) we applied a previously validated ranking approach and a correction for biological and batch effects using the edgeR statistical workflow [31, 32]. By employing the Ranking Index (RI) methodology to identify the differentially accessible regions (DARs), defined as CARs with a differential ranking index (RI) of their normalized CPMs between HBV and MOCK samples over a defined threshold of 20 (see online Supplementary Information), we revealed an average of ~ 4000 and ~ 7500 DARs at 2 h and 72 h p.i., respectively (Fig. 1c), with a relatively balanced number of up-regulated (i.e., regions with increased accessibility) and down-regulated (i.e., regions with decreased accessibility) regions in HBV vs MOCK samples (Figure S3b). The unsupervised hierarchical clustering analysis of these DARs confirms the impact of productive HBV infection on the host chromatin accessibility (Fig. 1d) with less prominent changes at early times post-infection (Fig. 1d; Figure S3c). Taken together these results indicate that HBV infection is associated with substantial chromatin remodeling.

### Chromatin remodeling in productively HBV-infected hepatocytes

**Fig. 2:**
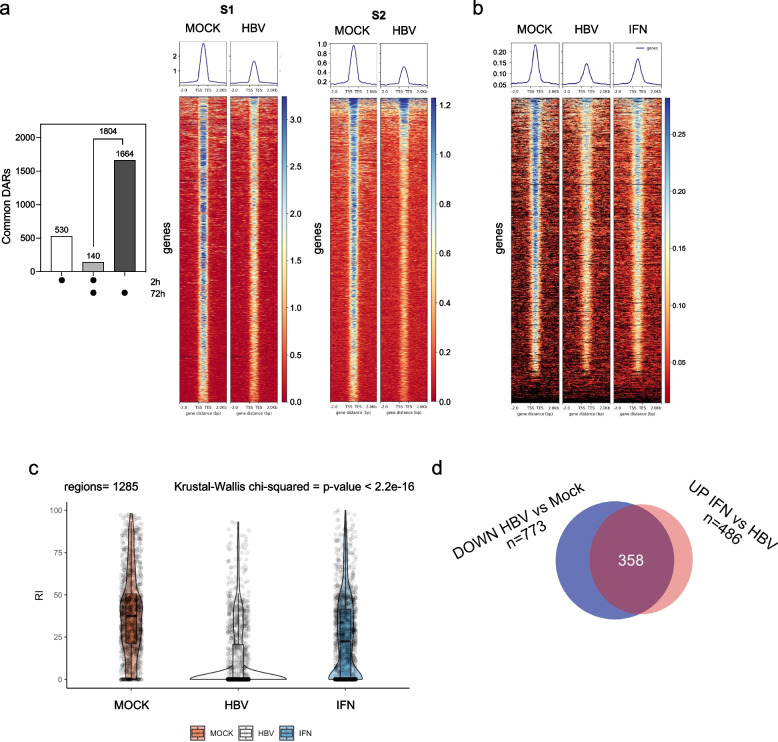
Chromatin remodeling in productively HBV-infected PHHs. **a** Chromatin accessibility in HBV-infected PHHs. Left panel: Differentially accessible regions (DARs) common to the biological (*N* = 2) and technical replicates (*N* = 2) at 2 h and 72 h post-infection (p.i.). The number of common DARs increases from 2 to 72 h p.i. (670 and 1804 respectively; *N* = 4 at each time point), with a small proportion of regions (*n* = 140) showing differential accessibility at both time points. Right panels: ATAC-seq density profiles of the 1804 DARs at 72 h p.i. in MOCK- and HBV-infected PHHs. S1 = donor 1; S2 donor 2. Intensities are represented with a color scale from blue (more accessible chromatin) to red (less accessible chromatin). **b** ATAC-seq density profiles at 72 h p.i. in MOCK, HBV-infected and IFN-treated HBV-infected PHHs (2 replicates from a third donor). Intensities values are represented as in a). **c** Violin plot depicting the ranking index (RI) distribution for the 1285 detected ATAC-seq peaks across MOCK, HBV, and IFN conditions, with two biological replicates per group. The RI values were generated as described in the supplementary data. A Kruskal–Wallis test was conducted to assess significant differences in RI distributions between the three groups. **d** VENN Diagram showing the intersection of the 773 ATAC-seq DARs down-regulated (i.e., displaying a reduced accessibility) in response to HBV infection with the 486 ATAC-seq DARs up-regulated by IFN treatment in HBV infected cells

To refine the analysis of host chromatin remodeling associated with productive HBV infection, we focused on the 1804 DARs modulated at 72 h p.i. in all biological and technical replicates that we defined as *common DARs*. We believe these are the most relevant chromatin sites to consider in that their chromatin accessibility is consistently modulated by HBV infection in all donors/samples (i.e., independently of the individual pre-existing chromatin landscape, see Fig. 1b) (Fig. 2a, *left panel*). The density profiles of these regions show that the prevalent chromatin remodeling effect imposed by HBV infection is a reduction of chromatin accessibility at selected sites (Fig. 2a, *middle and right panel*; Figure S4a).

To further assess the relation between productive HBV infection and the observed changes in chromatin accessibility, we performed additional ATAC-seq experiments treating HBV-infected PHHs with interferon-$\alpha$ (IFN$\alpha$) to inhibit both HBV RNAs transcription from the cccDNA (total HBV RNAs and 3.5 kb HBV RNAs) and downstream HBV capsid replication (HBV DNA) (Figure S4b). As shown in Figure S4c, all non-infected PHH samples showed a strong IFN response with the activation of several ISGs [10], confirming that PHHs are responsive to exogenous IFN$\alpha$. ATAC-seq analysis were performed at 72 h (p.i.) in MOCK-treated, HBV-infected, and IFN-treated (1000 IU/mL) HBV-infected PHHs. The density profiles (compare *right panel* vs *left* and *middle panels* in Fig. 2b) and the corresponding Violin plots (Fig. 2c) of the ranking index (RI) distribution for the 1285 ATAC-seq DARs detected across the MOCK, HBV, and IFN conditions, show that IFN $\alpha$ restores in part the chromatin accessibility changes imposed by HBV. Notably, 773 DARs were down-regulated in the HBV samples vs MOCK and 486 were up-regulated in the HBV-infected IFN-treated samples vs HBV infected samples. The intersection of the ATAC-seq DARs down-regulated in response to HBV infection with those up-regulated by IFN in HBV-infected cells shows that IFN treatment restores chromatin accessibility at 358 differentially accessible regions, representing ~ 45% of all HBV down-regulated DARs and > 70% of the DARs up-regulated by IFN vs HBV infected cells (Fig. 2d). Although we cannot formally exclude that some of the DARs down-regulated in HBV infected cells recover their chromatin accessibility because of a direct effect of IFN and not due to the relief of HBV activity mediated by the antiviral effect of IFN, altogether, these results support the notion that the partial restoration of the chromatin accessibility observed after IFN treatment of HBV-infected PHHs is indeed related to the IFN-mediated inhibition of viral replication.

**Fig. 3:**
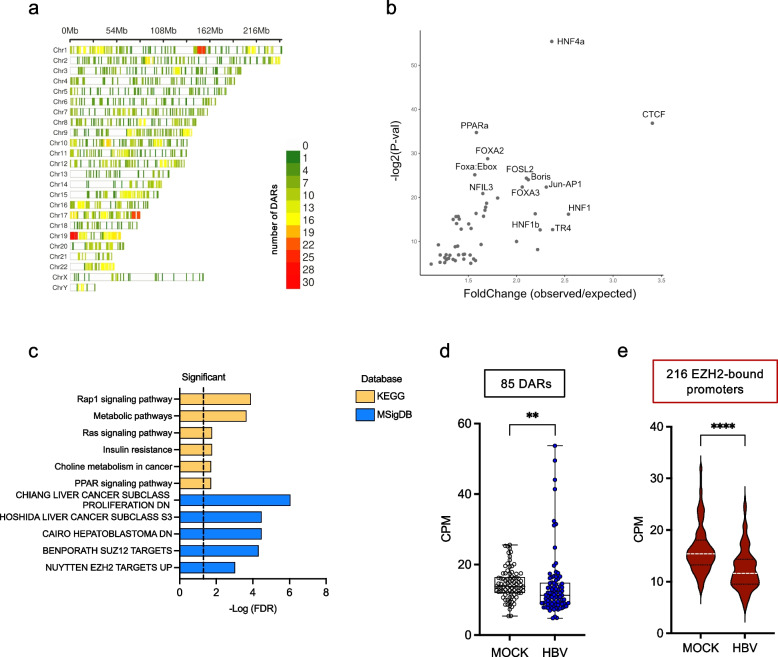
Characterization of chromatin differentially accessible regions in productively HBV-infected PHHs. **a**) Chromosome localization of the 1804 common DARs identified at 72 h p.i.. The color scale represents the number of DARs localized in each 10 MBps genomic regions. Three different hotspots were identified on chromosomes 1 (35 DARs), 17 (26 DARs), 19 (36 DARs). **b**) Motif Enrichment Analysis (MEA) of DNA-binding transcription factors (TFs) associated with the genomic sites showing differential accessibility after HBV infection (1804 common DARs). Names are indicated for the top significant TFs. **c**) Pathway enrichment analysis on the 1537 Differentially Accessible Genes (DAGs) potentially affected upon HBV infection using the ShinyGO 0.76 tool. Top significant KEGG pathways (Release 86.1; yellow bars) and Curated Molecular Signature Database (MSigDB) modules (Release 86.1; blue bars) enriched at 72 h p.i are sorted by –Log (FDR) values. Fold enrichment ranks the relative number of genes included in each enriched pathway (see Table S4). **d**) CPM distribution for the 85 DARs, corresponding to 81 DAGs that are enriched in the MSigDB, NUYTTEN EZH2 targets UP gene set in MOCK and HBV-infected PHHs (Table S5). **e**) CPM distribution for 216 regulatory regions (−5000/+ 5000 bp from TSS) in 214 genes showing EZH2 recruitment in vivo by ChIP-Seq in HepG2-NTCP cells (Table S6). ** = *p* < 0,001

The localization of the 1804 common DARs identified at 72 h p.i. shows that, although HBV-induced chromatin accessibility changes occur throughout the genome and in all chromosomes, 3 hotspots can be identified in Chromosomes 1, 17 and 19 (Fig. 3a; Table S3). As expected, the DARs localized in these hotspot regions tend to close following infection (Figure S4 d). Next, Motif Enrichment Analysis (MEA) was utilized to identify transcription factors (TFs) potentially responsible for regulating the expression of genes associated with the 1804 DARs in HBV-infected hepatocytes. Notably, these TFs included HNF1 A and HNF4 A, which are known to play a critical role in liver cell identity, as well as NFIL3 and HNF1b, involved in glucose metabolism, and PPARa, FOXA2, and FOXA3, related to lipid metabolism (Fig. 3b).

To correlate HBV-induced host chromatin remodeling to biological functions, we then annotated the 1804 *common DARs* by assigning the closest gene and identified 1537 Differentially Accessible Genes (DAGs) potentially affected in HBV productively infected cells. Using these 1537 DAGs, we performed a pathway enrichment analysis to interrogate the KEGG (Kyoto Encyclopedia of Genes and Genomes) Pathway bioinformatic resource, that maps molecular interaction, reactions and relation networks, and the MSigDB (Molecular Signatures Database), collections of gene data sets, molecular signatures and gene expression signatures. KEGG Patways enrichment analysis highlights the enrichment, at 72 h p.i., in genes involved in cell metabolism (e.g., metabolic pathways, insulin resistance, PPARa signaling) (Fig. 3c; Table S4). Of note, PPAR$\alpha$ binds the HBV minichromosome and activates cccDNA transcription and HBV replication [33]. We also found an enrichment of genes involved in *RAP-1 signaling*, *Ras signaling* and *Choline metabolism in cancer* (Fig. 3c). The interrogation of MSigDB database [34] also showed the enrichment at 72 h p.i. of genes belonging to modules related to the PRC2 (Polycomb Repressive Complex 2) chromatin complex, its components and its targets (Fig. 3c). Notably, most of the 81 DAGs (from 85 DARs) that are enriched in the *MSigDB, NUYTTEN EZH2 targets UP* gene set (see Fig. 3c) display a reduced chromatin accessibility after HBV infection (Fig. 3d*;* Table S5). We could also show that 216 regulatory regions in the 214 genes bound in vivo by EZH2 in HepG2-NTCP cells have reduced chromatin accessibility (Fig. 3e*;* Table S6). Thus, even though individual PHH donors present a specific chromatin accessibility profile by ATAC-seq, we could identify a common set of DARs/DAGs affected by HBV infection and related to liver metabolism and PRC2-targets.

### Integration of chromatin accessibility and transcriptomic profiles

**Fig. 4:**
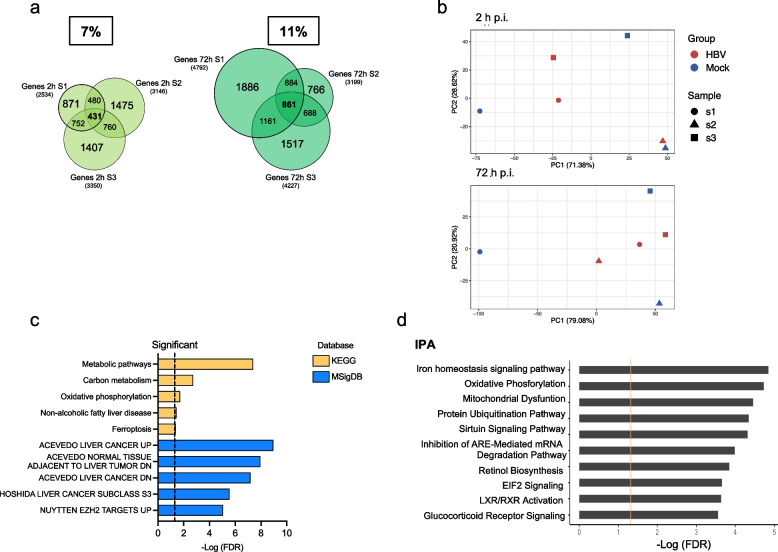
Transcriptional changes in HBV-infected PHHs. **a**) Venn diagrams of RNA-seq Differentially Expressed Genes (DEGs) at 2 h (light green) and 72 h (dark green) p.i. in three independent experiments (S1 = donor 1; S2 = donor 2 and S3 = donor 3). The pipeline for the identification of Differentially Expressed Transcripts (DETs) and the definition of a unique list of Differentially Expressed Genes (DEGs) is detailed in the Supplementary information. **b**) Principal Component Analysis (PCA) of S1, S2 and S3 RNA-seq global transcriptomic profiles at 2 h p.i. (upper panel) and 72 h p.i. (lower panel). For each time point the reference space of the PCA has been computed using the Mock samples (blue dots, triangles and squares), following by the projection of the infected samples (red dots). At 72 h p.i. we can appreciate a more evident clustering of the transcriptomic profiles upon HBV infection (red dots, triangles and squares). **c**) KEGG pathways (Release 86.1; yellow bars) and HALLMARK MSigDB modules significantly enriched from the Differentially Expressed Genes (DEGs) at 72 h p.i. (ShinyGO 0.76 tool) are sorted by–Log (FDR) values. Fold enrichment ranks the relative number of genes included in each enriched pathway (see Table S7). **d**) Ingenuity Pathways Analysis (IPA) of the DEGs identified at 72 h p.i

Next, we integrated the results of ATAC-seq (DAGs) and RNA-seq (DEGs) analysis in HBV-infected PHHs. To this aim, RNA-seq were performed in three different donors before (MOCK) and after HBV infection at 2 and 72 h. Despite the different basal transcriptomic profiles, the number of common DEGs among the three donors increased over time post-infection (431/7% at 2 h vs 861/11% at 72 h) (Fig. 4a). These results indicate that the chromatin remodeling imposed by HBV infection and its translation into a transcriptional reprogramming of infected hepatocytes converge over time towards a common phenotype (Fig. 4b). DEGs pathway enrichment analysis by KEGG, MSigDB (Fig. 4c; Table S7) and IPA (Ingenuity Pathway Analysis) tool (Fig. 4d) showed a strong modulation of pathways involved in cell metabolism (e.g., metabolic pathways, carbon metabolism, oxidative phosphorylation, glucocorticoid receptor signaling, LXR/RXR activation) and liver cancer (Fig. 4c-d). Additional enriched pathways include epigenetic effectors (e.g., Sirtuins signaling, Ezh2 targets) (Fig. 4c-d), and iron metabolism (Fig. 4d).

**Fig. 5:**
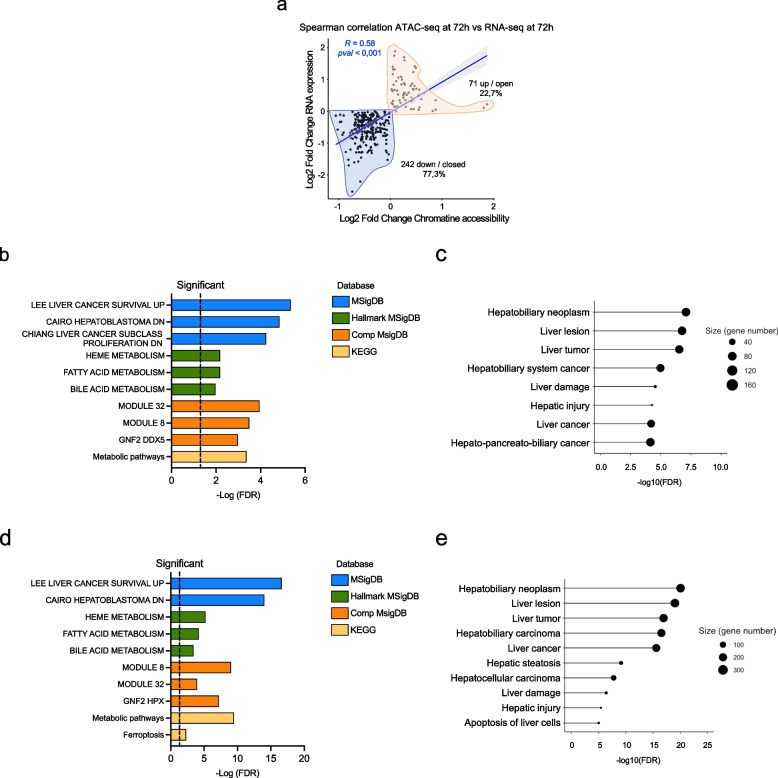
Integration of ATAC-seq and RNA-seq data. **a**) Log2 Fold change correlation analysis (Spearman R test) in the 313 genes with significant and concordant changes in both ATAC-seq (DARs) and RNA-seq (DEGs) (e.g., higher chromatin accessibility corresponding to higher expression levels in HBV infected cells, and vice versa) at 72 h p.i. (*co-regulated genes*). The 242 genes (77.3%) showing a reduced chromatin accessibility and reduced expression after HBV infection (negative Log2 fold change values) are shadowed in light blue. The 71 genes (22.7%) with increased chromatin accessibility and increased expression after HBV infection (positive Log2 fold change values) are shadowed in light red. **b**) MSigDB (blue bars), Hallmark MSigDB (green bars), Computational (Comp) MSigDB (orange bars) and KEGG (dark yellow bars) enrichment analysis (ShinyGO 0.76 tool) for21 the 313 concordant ATAC-seq (DARs) and RNA-seq (DEGs) genes at 72 h p.i. (see Table S8). **c**) Ingenuity Pathways Analysis (IPA) of the 313 concordant ATAC-seq (DARs) and RNA-seq (DEGs) genes at 72 h p.i. **d**) MSigDB (blue bars), Hallmark MSigDB (green bars), Computational (Comp) MSigDB (orange bars) and KEGG (dark yellow bars) enrichment analysis (ShinyGO 0.76 tool) for 643 genes impacted by HBV infection both in ATAC-sec (DARs) and RNA-seq RNA-seq (DEGs) (comodulated genes) at 72 h p.i. (see Table S9). **e**) Ingenuity Pathways Analysis (IPA) of the 643 *co-modulated genes* with significant changes in both RNA-seq (DEGs) and ATAC-seq (DARs) profiles at 72 h p.i

The integration of the ATAC-seq and RNA-seq analysis did not allow to identify direct and univocal correlations between all changes in chromatin accessibility and gene expression at the different times post-infection. Indeed, temporal or cell type-specific dissociations between chromatin accessibility and transcription have been reported [35]. Nevertheless, we identified 313 *co-regulated genes* that had significant and concordant changes in both RNA-seq (DEGs) and ATAC-seq (DARs) profiles (e.g., higher chromatin accessibility corresponding to higher expression levels in HBV infected cells, and vice versa) at 72 h p.i. (Fig. 5a). Among these concordant genes 77.3% showed negative Log2 fold change values, in line with the notion that the prevalent, but not exclusive, host response early after HBV infection is a reduced chromatin accessibility with transcriptional repression. MSigDB and KEGG analysis of these 313 genes confirmed the enrichment in genes involved in liver metabolic and cancer pathways (Fig. 5b; Table S8). These results were further supported by IPA analysis (Fig. 5c).

Since chromatin accessibility measures the potential for transcription, indicating priming for future expression or a remnant ‘scar’ of past transcription [35], direct correlation with transcriptional profiles (e.g., at the same time points after HBV infection), may overlook asynchronous, yet relevant, gene expression changes prompted by HBV-induced changes in chromatin accessibility. Thus, we enlarged our analysis to all the 643 genes that were modulated by HBV infection both in ATAC-sec and RNA-seq (*comodulated* genes) at 72 h p.i.. MSigDB and KEGG analysis show again an enrichment in genes involved liver cancer (Comp MSigDB modules 8 and 55) and liver metabolism (e.g., xenobiotic, fatty acids, and bile acids metabolism) (Fig. 5d; Table S9) and also unveiled an involvement of iron metabolism in the cellular response to HBV infection (module GNF2 HPX) (Fig. 5d). The IPA of the 643 *comodulated* genes shows an enrichment in genes involved liver damage as well as in liver cancer (Fig. 5e).

### HBV infection modulates iron metabolism to promote its replication

**Fig. 6:**
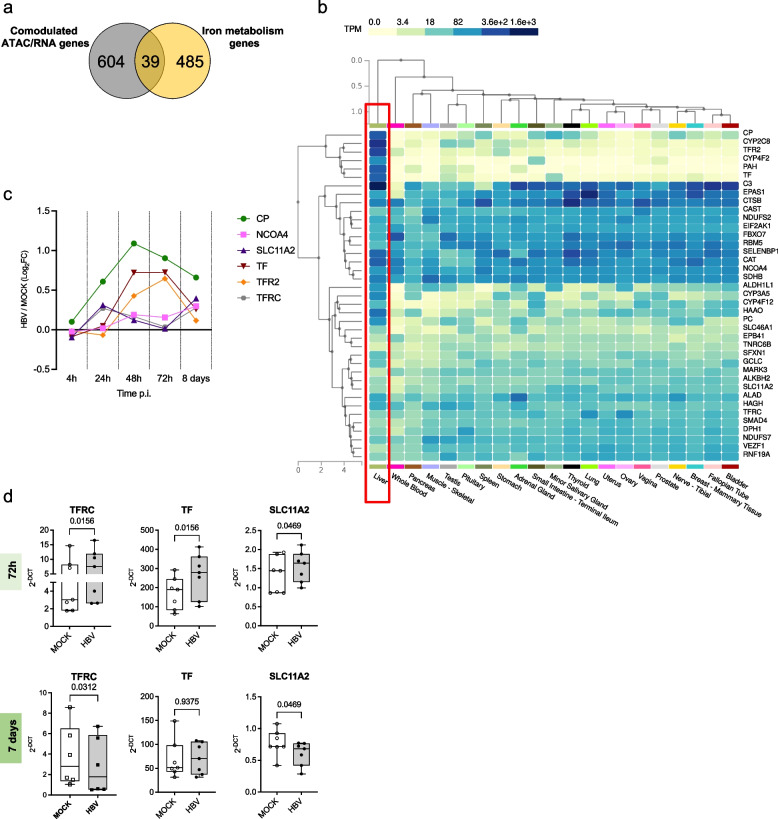
Modulation of iron metabolism-related genes after HBV infection. **a**) Venn diagram showing the intersection between the 643 RNA-seq (DEGs) and ATAC-seq (DAGs) comodulated genes and iron metabolism genes (524 genes from 23 iron-related MSigDB gene sets; see the Supplementary Information and Table S10). **b**) Heatmap of RNA-seq median Transcripts Per Million (TPM) values, stratified by organ, in the GTEx project database for the 39-iron metabolism-related genes comodulated in RNA-seq (DEGs) and ATAC-seq (DAGs) after HBV infection (liver iron signature). The hierarchal clustering analysis indicate a clear separation between liver (red rectangle) and other tissues. **c**) Expression of the 6 genes involved in iron uptake (CP, TF, TFR2 and TFRC) and iron homeostasis (NCOA4, SLC11 A2) present in the liver iron signature in a RNA-seq dataset of HBV infected hepatocytes [10]. Results are expressed as Log2 Fold Changes (Log2 FC) in HBV vs MOCK conditions at the indicated times p.i. CP, TF, TFR2, TFRC, NCOA4, SLC11 A2. **d**) Quantitative Real-Time PCR analysis of 3 iron-uptake/homeostasis genes in the liver iron signature (TF, TFRC and SLC11 A2) in MOCK and HBV-infected PHHs from seven independent experiments. Data are expressed as the 2-$\Delta$CT of target genes normalized over the GUSb housekeeping gene. Whiskers represent the minimum and maximum values. *p*-values were calculated using the Wilcoxon matched-pairs signed rank test

Next, we intersected the 643 ATAC-seq/RNA-seq *comodulated genes* with a list of 524 genes compiled from 23 MSigDB iron-related gene sets (see online Supplementary Information). We identified 39 iron metabolism-related genes whose expression is modulated by changes in chromatin accessibility after HBV infection (Fig. 6a; Table S10). Notably, these 39 genes are highly expressed in the liver as compared to other normal tissues in the GTEx database (https://gtexportal.org/home/) (Fig. 6b). Among the 39 liver iron signature genes, 6 are involved in iron uptake (*CP, TF, TFR2* and *TFRC*) and iron homeostasis (*NCOA4, SLC11 A2*). Their upregulation was confirmed in an independent RNA-seq dataset of HBV infected hepatocytes [10] (Fig. 6c). Additional, independent HBV infection experiments in PHHs confirmed the overexpression of *TFRC*, *TF* and *SLC11 A2*, at 72 h p.i. followed by a return to baseline or a decrease at 7 days (Fig. 6d).

**Fig. 7:**
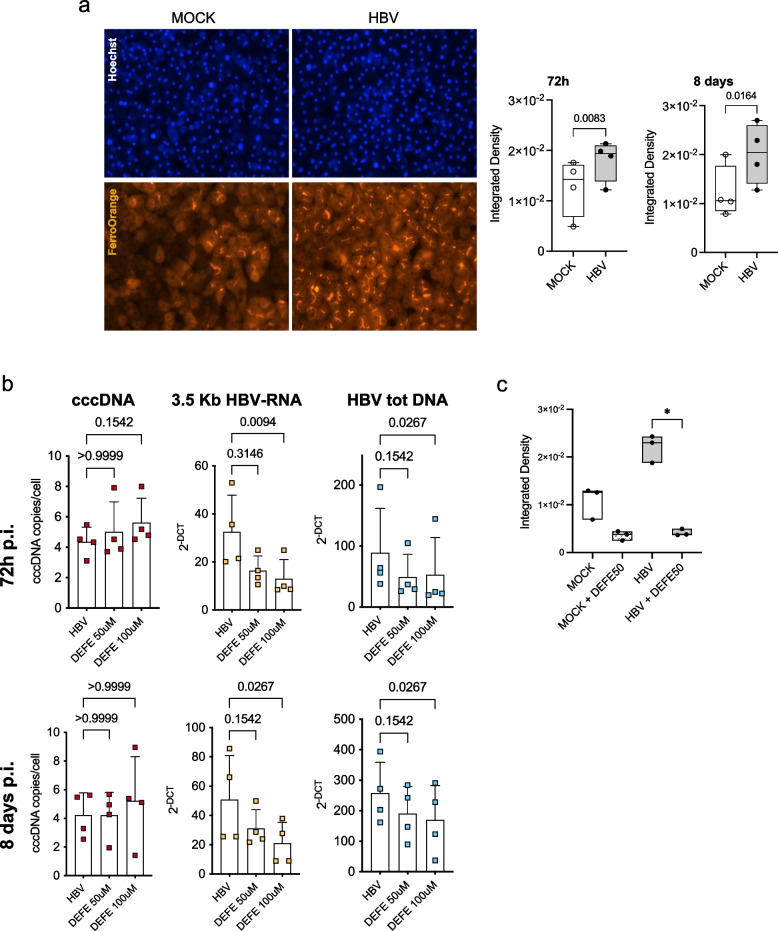
HBV infection increases iron uptake to promote viral replication. **a**) Iron uptake increases after HBV infection. Left panels: Nuclei (Hoechst) and free Fe + 2 ions (FerroOrange) staining in MOCK and HBV-infected PHHs at 8 d p.i. Magnification 20x. Right panels: Integrated density of Fe + 2 ions staining (*n* = 4 experiments) at 72 h p.i. (right upper panel) and 8 days p.i. (right lower panel). Whiskers represent the minimum and maximum point. *p*-values were calculated using Student's paired t-test. **b**) Free available Fe^+2^ is required for HBV replication. Quantification of covalently closed circular DNA (cccDNA) by PCR (left panels), 3.5 Kb HBV-RNA species by RT-PCR (middle panels) and total HBV DNA by PCR (right panels) in HBV-infected PHHs untreated or treated with 50- or 100 µM Deferasirox (DEFE) at 72 h (upper panels) and 8 days (lower panels). Error bars represent standard deviations (SD) of four independent experiments. *p*- values were calculated using the Friedman test with Dunn’s correction for multiple comparison tests. **c**) Integrated density of Fe^+2^ ions staining (*n* = 3 experiments) at 8 days p.i.. Nuclei (Hoechst) and free Fe^+2^ ions (FerroOrange) staining in MOCK, MOCK treated (DEFE50 uM), HBV-infected and HBV-infected and treated (DEFE50 uM) PHHs at 8 d p.i. Magnification 20x. *p*-values were calculated using Kruskal–Wallis test

Many viruses enhance Fe^+2^ uptake in the infected cells to promote their own replication [36]. We found a significant increase in iron uptake in PHHs upon HBV infection (Fig. 7a). Next, we asked whether the increase in iron cellular load may have an impact on HBV replication. Treatment of HBV-infected PHHs with Deferasirox (DEFE), that chelates non-transferrin bound iron (free iron) as well as iron in transit between transferrin and ferritin (labile iron pool), reduces viral replication with a decrease in HBV 3.5 kb RNA species, including HBV pgRNA, and HBV DNA without any significant impact on the cccDNA nuclear pool (Fig. 7b) and in the absence of any significant impact on cell viability (Figure S5a). Notably, DEFE strongly reduces iron uptake in HBV-infected PHHs (*p* = 0.038) and to a lesser extent in MOCK PHHs (Fig. 7c, Figure S5b), indicating that the inhibitory effect of DEFE on HBV replication is most likely linked to its effects on iron metabolism. These results show, in a relevant in vitro HBV infection model, that HBV alters iron homeostasis early after infection and requires free available Fe^+2^ for its replication.

### The early transcriptional changes in HBV-infected hepatocytes persist in CHB patients

**Fig. 8:**
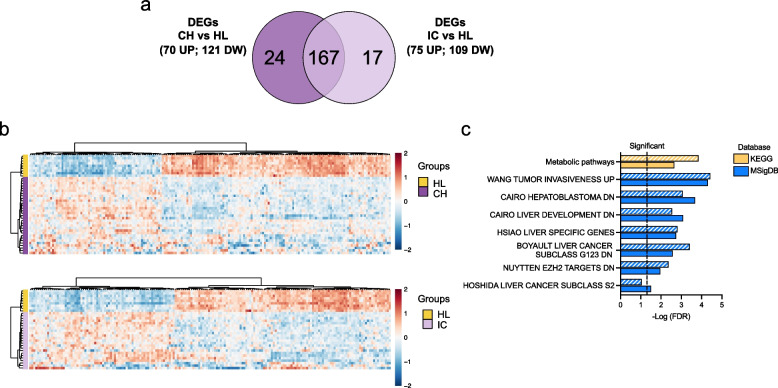
The transcriptional signature of HBV-infection in vitro persists in CHB patients. **a**) Venn diagram of the 208 genes differentially expressed in the RNA-seq from CHB patients [32] and *co-regulated* in ATAC-seq/RNA-seq from HBV-infected PHHs. Most of the genes differentially expressed in HBe pos and HBe neg chronic hepatitis (CH) vs non-HBV infected healthy livers (HL) and HBV inactive carriers (IC; e.g., HBe neg chronic infection (CI)) vs HL shows that most DEGs are shared (*n* = 167), 24 DEGs unique to HBe pos and HBe neg CH groups and 17 DEGs exclusive of the HBe neg CI patients. **b**) Upper panel: Heatmap of the 191 ATAC-seq/RNA-seq *co-regulated* genes that are differentially expressed in CH patients (HBe pos and HBe neg CH; purple) vs non-HBV infected healthy livers (HL, dark yellow). Lower Panel: Heatmap of the 184 ATAC-Seq/RNA- seq *co-regulated* genes that are differentially expressed in HBV inactive carriers (IC; e.g., HBe neg chronic infection (CI); light purple) vs non-HBV infected healthy livers (HL, dark yellow). Normalized counts are represented with a color scale from blue (negative) to red (positive). **c**) KEGG pathways (Release 86.1; yellow bars) and HALLMARK MSigDB modules significantly enriched from the 191 genes differentially expressed in CH vs HL (full bars) and the 184 genes differentially expressed in IC vs HL (striped bars) (ShinyGO 0.76 tool) and sorted by–Log (FDR) values. Fold enrichment ranks the relative number of genes included in each enriched pathway (see Tables S12 and S13)

To investigate the potential clinical impact of the early changes we described in HBV infected hepatocytes in vitro, we interrogated three independent RNA-seq data sets from chronically HBV infected patients [37] and HBV-related HCCs. Out of the 313 *co-regulated* genes in our HBV-infected PHHs, 191 were differentially expressed (121 down-regulated; 70 up-regulated; Table S11) in liver biopsy samples of HBV chronic hepatitis (CH) patients (CH HBe pos, *N* = 15 + CH HBe neg, *n* = 16) as compared to non-HBV infected healthy liver (HL) samples (*N* = 9) (Fig. 8a; Fig. 8b, *upper panel*). KEGG and MSigDB analysis of these 191 genes confirmed the enrichment of cancer and liver metabolic pathways (Fig. 8c, *filled bars*; Table S12). Of note, 184 out of the 313 *co-regulated* genes were also differentially expressed (109 down-regulated; 75 up-regulated; Table S11) in the liver of HBV inactive carriers (IC, e.g., HBe neg chronic infection (CI); *n* = 23) as compared to the HL samples (Fig. 8a; Fig. 8b, *lower panel*). These gene enriched the same KEGG and MSigDB pathways (Fig. 8c, *striped bars*; Table S13). Most of the overall 208 genes differentially expressed in CHB and *co-regulated* in our ATAC-seq/RNA-seq were shared (*n* = 167) between the different CHB categories whereas 24 were unique to CH group (HBe pos CH and HBe neg CH) and 17 exclusives of the HBe neg CI patients (Fig. 8a).

We also found that of the 39-iron signature genes modulated in HBV-infected PHHs (see Fig. 6b) 30 genes (24 down-regulated; 6 up-regulated; Table S14 were differentially expressed in the liver of CH patients vs HL (Figure S6a) 29 (23 down-regulated; 6 up-regulated) in the liver of IC patients vs CH patients (Figure S6b). Altogether these results indicate that the chromatin accessibility and transcriptional changes imposed by HBV early after infection may persist as an epigenetic scar in chronic HBV patients to a large extent independently from the levels of viral replication and disease activity in the chronic phase.

**Fig. 9:**
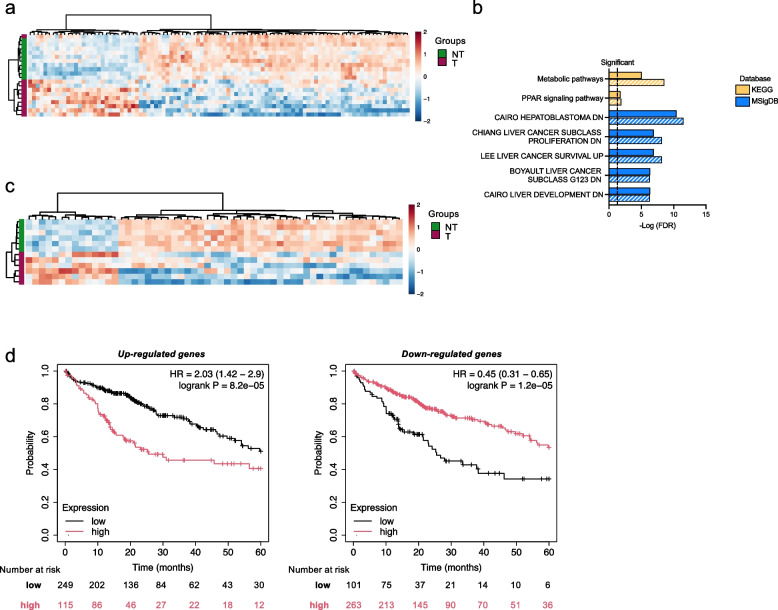
Early transcriptional changes imposed by HBV-infection in vitro persist in HBV-related HCC patients. **a**) Heatmap of the 100 ATAC-Seq/RNA-seq *co-regulated* genes that are differentially expressed (71 down- and 29 up-regulated) in the *in house* Lyon cohort of matched HBV-related HCCs (T; magenta) and Non-Tumor (NT; green) tissues (*n* = 10). Normalized counts are represented with a color scale from blue (negative) to red (positive). **b**) KEGG pathways (Release 86.1; yellow bars) and HALLMARK MSigDB modules (blue bars) significantly enriched from the 100 genes differentially expressed in the *in-house* Lyon cohort of matched HBV-related HCC samples (T vs NT; full bars) and the 57 genes differentially expressed in TCGA-LIHC subset of matched HBV-related HCCs (T vs NT; striped bars) (ShinyGO 0.76 tool) and sorted by–Log (FDR) values. Fold enrichment ranks the relative number of genes included in each enriched pathway (see Tables S15 and S11). **c**) Heatmap of the 57 ATAC-Seq/RNA-seq *co-regulated* genes that are differentially expressed (43 down- and 14 up-regulated) in the TCGA-LIHC subset of matched HBV-related HCCs tissues (*n* = 6; T: magenta and NT; green). Normalized counts are represented as in a). **d**) Kaplan–Meier curves generated comparing the 14/57 up-regulated genes (left panel) and the 43/57 down-regulated genes (right panel) in the whole TCGA-LIHC

Next, we performed similar analysis using 2 independent HBV-related HCC RNA-seq datasets. In a in *house* cohort of matched HBV-related HCCs (T) and Non-Tumor (NT) tissues (*n* = 10) and Healthy Liver (HL) samples (*n* = 5) (Lyon cohort), we found that 100 out of the 313 ATAC-seq/RNA-seq *co-regulated* genes were differentially expressed (71 down-regulated and 29 up-regulated; Table S11) in the T vs NT tissues (Fig. 9a) and were enriched in liver cancer and metabolism genes in the MSigDB and KEGG analysis, respectively (Fig. 9b; Table S15). We also found that 55 out of the 313 ATAC-seq/RNA-seq *co-regulated* genes were differentially expressed (42 down-regulated and 13 up-regulated; Table S11) when the T tissues were compared to HL tissues (Figure S7a). Notably, these 55 DEGs were also enriched in liver cancer and metabolism genes in the MSigDB and KEGG analysis and 45/55 genes overlapped with the DEGs from the comparison of T vs NT tissues (Figure S7b and Table S16). Similar results were obtained when we analyzed the 6 paired tumor (T) and non-tumor (NT) HBV-related HCC tissues from TCGA LIHC database (https://www.cancer.gov/tcga) with 57 DEGs (43 down-regulated and 14 up-regulated; Fig. 9c and Table S11). Fifty-one of these 57 DEGs overlapped with the DEGs from the Lyon cohort, and they also enriched in liver cancer and metabolism genes (Fig. 9b and Table S17). Finally, a high expression of the 14 up-regulated genes was associated in the Kaplan Meyer's analysis with lower survival in the entire TCGA LIHC cohort (Fig. 9d, left panel) and, conversely, a higher expression of the 43 down-regulated genes was associated with a better survival (Fig. 9d, right panel).

These results further support the notion that the early changes in chromatin accessibility and transcription imposed by HBV infection may persist and favor the development/selection of a pro-neoplastic phenotype.

We then investigated whether the 39 iron signature genes identified in HBV-infected PHHs and in chronic hepatitis B patients was also modulated in the HBV-related HCC from the TCGA and the Lyon cohort. As shown in Figure S8, 17 genes (15 down-regulated; 2 up-regulated; Table S14) were differentially expressed in the paired T vs NT liver tissues from the HBV-related HCCs of the TCGA dataset (Figure S8a) and 19 (18 down-regulated; 1 up-regulated) in HCCs from the Lyon HBV HCC cohort (Figure S8b). Of note, the number of iron signature genes deregulated in HBV-related HCCs is lower that in HBV-infected PHHs and in CH patients and, more importantly, the genes involved in iron uptake are under-represented.

Finally, we explored whether the pro-oncogenic pathways modulated by HBV early after infection and persisting in chronic HBV patients and HBV-related HCCs are specific for HBV, or they are part of a broader HCC phenotype. To this aim we checked how many of the 313 *genes co-regulated* in HBV-infected PHHs were modulated in 18 HCV-related HCCs from the Mongolian HCC cohort [38] and in 11 non viral (alcohol- and/or MASH-related) HCCs from the TCGA LIHC database. We found 121/313 (38.6%) genes deregulated in HCV-related HCCs and 94/313 (30.0%) in non-viral HCCs. Notably, 44 genes were shared among the 3 HCC etiologies, representing from 36% (HCV-related HCCs) to 44% (HBV-related HCCs) and 46% (non-viral HCCs) of the total deregulated genes for each etiology. Altogether these results indicate that the genes modulated at early stages of HBV infection and differentially expressed in HBV-related HCCs comprise both genes specific of the HBV etiology as wells as genes linked to oncogenic pathways shared with another HCC etiology as well as a core of liver cancer-related genes independent from the HCC etiology.

## Discussion

Our understanding of the quality and the extent of the impact of HBV infection on the host cell transcriptome is still limited. Indeed, the common notion of HBV being a"stealth virus"must be restricted to the lack of class I interferon induction due to the very limited sensing of the virus by the innate immune system at the time of acute infection. Although increasing evidence indicate that acute infection is accompanied, at least in in vitro infection systems, by a measurable impact on the host cell transcriptome [15–17] the co-existence of long-term chronic inflammation and viral replication in CHB patients greatly limits the identification of the direct contribution of the virus to the transcriptomic changes observed in untreated CHB patients [18, 19].

In eukaryotic cells, transcriptional activation is linked to the disruption of nucleosome organization at regulatory elements and chromatin remodeling. Thus, we sought to perform an unbiased genome wide evaluation of chromatin accessibility by ATAC-seq, coupled with high throughput transcriptomic profiling by RNA-seq in HBV-infected PHHs, a relevant model of HBV infection in vitro. We have chosen to use PHHs from different donors to mimic what occurs in natural infections where HBV infects multiple subjects with different epigenomes. We found that, although each donor exhibits a unique repertoire of ATAC-seq peaks, over time the landscape tends to converge when HBV infection is fully established with a pool of cccDNA that is actively transcribed to support viral genome replication and viral protein production. The prevalent, but not exclusive, change imposed by the virus is a global reduction of chromatin accessibility. Notably, a short treatment with IFN$\alpha$, that inhibits viral replication at multiple steps including transcription from the cccDNA [39], prevents at least in part these changes. In this respect, it is worth to underline that IFN$\alpha$ remains to date the only drug capable to lead to a functional cure with a finite treatment in some CHB patients [5]. It will be important in the future to confirm whether IFN$\alpha s$ induces chromatin changes like those we observed in vitro also in the liver of patients treated in the context of a long-lasting chronic infection.

ATAC-seq detects changes in the accessibility of native chromatin that are not necessarily linked to actual transcription but as already mentioned, may indicate priming for future transcription, or may reflect past transcription as a persistent scar or just because chromatin has not yet closed-up [35]. Indeed, temporal dissociations between chromatin accessibility and transcription have been reported, and this might be even more relevant in the highly dynamic context of a viral infection in vitro. Pathways that may be relevant for HBV replication and HBV pathogenesis were identified as modulated by HBV in ATAC-seq and/or RNA-seq analysis, but we could not demonstrate a direct and univocal correlation between chromatin accessibility and gene expression. Examples include Ezh2 targets (e.g., genes modulated by the PRC2 (Polycomb Repressive Complex 2) chromatin complex responsible for the deposition of the repressive histone mark K27 me3) and ferroptosis. Ezh2 activity has been related to HBV replication and HCC [40, 41]. Ferroptosis has not been previously linked to HBV infection per se*,* but it has been increasingly involved in HCC development/progression and in the response to treatments [42, 43].

Despite the above-mentioned limitations, the integration of ATAC-seq and RNA-seq allowed us to show that HBV infection modulates three main sets of biological functions. First, HBV impacts on several liver metabolic pathways (e.g., xenobiotic, fatty acids, cholesterol and bile acids metabolism and carbon metabolism). Of note, among the putative transcription factors that may bind chromatin regions whose accessibility is modified by HBV infection, and hence potentially capable to regulate the genes associated with these regions, we found NFIL3 and HNF1b, PPARa, FOXA2, and FOXA3 that are involved in glucose and lipid metabolism. A second set of pathways and genes affected by HBV infection are related to liver injury and liver cancer.

The integrative analysis of ATAC-seq and RNA-seq results also allowed us to identify a *liver iron signature* of 39 genes whose expression is modulated by HBV through changes in chromatin accessibility at their regulatory regions and among them the upregulation of genes involved in iron uptake. Indeed, many viruses enhance Fe^+2^ uptake in the infected cells and promote their own replication [36] and we could show a significant increase of free iron in HBV-infected PHHs.

Due to technical limitations in the co-staining for iron with FerroOrange and HBc immunofluorescence we can not formally exclude that the observed increase in iron uptake may be mediated by factors that are released in response to hepatocytes infection and involve uninfected cells. Of note, a bystander effect has been evoked to explain that some of the gene expression changes induced by HBV infection of the human hepatocytes in liver humanized mice were mirrored in the mouse hepatocytes that cannot be infected by HBV [44]. However, the observation that chelation of both free iron and the labile iron pool by Deferasirox blunts the iron uptake response and inhibits cccDNA transcription and viral replication, indicates that the induction of iron uptake indeed occurs in HBV-infected PHHs and that HBV requires free iron for its replication and re-wires iron metabolism genes at the chromatin level to support its needs.

The potential clinical impact of the early changes observed in HBV-infected hepatocytes in vitro was confirmed by interrogating RNA-seq data sets from chronically HBV infected patients and HBV-related HCCs. About 2/3 of the genes *co-regulated* in ATAC-seq/RNA-seq in HBV-infected PHHs were also differentially expressed in CHB livers as compared to non-HBV infected healthy liver samples. Most of these differentially expressed genes were also similarly modulated in HBV inactive carriers (e.g., HBe neg chronic infections, CI) as compared to the healthy livers with a large overlap across the CHB categories. Only a minority of them were differentially expressed specifically in CHB patients or inactive carriers. Altogether these results indicate that the chromatin accessibility and transcriptional changes imposed by HBV early after infection persist in chronic HBV patients mostly independently from the levels of viral replication and disease activity in the chronic phase. Notably, between 15 and 30% of the ATAC-seq/RNA-seq *co-regulated genes* were also differentially expressed in HBV-related HCC tissues, confirming that the changes observed early after HBV infection not only persist but may also favor the development or the selection of a pro-neoplastic phenotype. Notably, the interrogation of additional datasets from HCV-related HCCs and from non-viral HCCs showed that the genes modulated at early stages of HBV infection and differentially expressed in HBV-related HCCs comprise both genes specific of the HBV etiology, genes linked to oncogenic pathways shared with another HCC etiology and a core of liver cancer-related genes independent from the HCC etiology. Our results are in line with previous observations made in HBV infected cells in vitro [16] and link these transcriptional changes to a direct impact of HBV infection on the host cell chromatin landscape. The concept of a pro-neoplastic imprinting imposed by a virus early after infection that may contribute to the development of a liver cancer many years later is supported by several studies in the context of HCV infection where an epigenetic scar is established early after acute infection, persists after eradication of HCV with direct antivirals and is conserved in the tumor tissue of patients that develop an HCV-related HCC [45, 46]. The observation that those genes modulated at the chromatin and transcriptional level by HBV infection and that are up-regulated or down-regulated in the HCC tissues bear a prognostic significance in HBV-related HCCs but also in HCCs of different etiology, further support the notion that the oncogenic potential of HBV is at work already early after infection and it is not limited to early integration of viral sequences into the host genome [47].

## Conclusions

Altogether our results show that HBV infection impacts on host cell chromatin landscape and specific transcriptional programs that include several liver metabolic pathways (e.g., xenobiotic, fatty acids, cholesterol and bile acids metabolism, carbon metabolism, iron metabolism) and liver cancer pathways. Notably, re-wiring of iron metabolism boosts viral replication early after infection. The chromatin accessibility and transcriptional changes imposed by HBV early after infection persist as an epigenetic scar in chronic HBV patients, to a large extent independently from the levels of viral replication and disease activity in the chronic phase. The modulation of genes involved in cancer-related pathways may favor the development or the selection of a pro-neoplastic phenotype and persists in HBV-related HCCs.

## Supplementary Information

Supplementary Material 1.

Supplementary Material 2.

Supplementary Material 3.

Supplementary Material 4.

Supplementary Material 5.

Supplementary Material 6.

Supplementary Material 7.

Supplementary Material 8.

Supplementary Material 9.

Supplementary Material 10.

Supplementary Material 11.

Supplementary Material 12.

Supplementary Material 13.

Supplementary Material 14.

Supplementary Material 15.

Supplementary Material 16.

Supplementary Material 17.

Supplementary Material 18.

Supplementary Material 19.

Supplementary Material 20.

Supplementary Material 21.

Supplementary Material 22.

Supplementary Material 23.

Supplementary Material 24.

Supplementary Material 25.

Supplementary Material 26.

## Data Availability

Data Deposition: - Database ATAC-Seq: GSE240183 study at: https://www.ncbi.nlm.nih.gov/geo/query/acc.cgi?acc=GSE240183; - Database RNA-Seq: GSE239860 study at: https://www.ncbi.nlm.nih.gov/geo/query/acc.cgi?acc=GSE239860; - Database HCC-HBV: GSE251942 study at: https://www.ncbi.nlm.nih.gov/geo/query/acc.cgi?acc=GSE251942. Additional data is provided within the supplementary information files.

## Abbreviations

ATAC: Assay for Transposase Accessible Chromatin
CARs: Chromatin Accessible Regions
CHB: Chronic Hepatitis B
CPM: Counts Per Million
DAGs: Differentially Accessible Genes
DARs: Differentially Accessible Regions
DEGs: Differentially Expressed Genes
DETs: Differentially Expressed Transcripts
MEA: Motif Enrichment Analysis
NGS: Next-Generation Sequencing
NUCs: Nucleos(t)ide analogs
PHHs: Primary Human Hepatocytes
PRRs: Pattern Recognition Receptor
RI: Rank Index
TF: Transcription Factor
TMM: Trimmed Mean of M values
TPM: Transcripts Per Million
TSS: Transcription Starting Site
