# Supplementary figures and images for: HBV-driven host chromatin accessibility changes affect liver metabolic pathways, iron homeostasis and promote a preneoplastic phenotype
Source: J Exp Clin Cancer Res. 2025 May 16;44:146. doi: 10.1186/s13046-025-03414-7 (PMC12082925; doi:10.1186/s13046-025-03414-7)

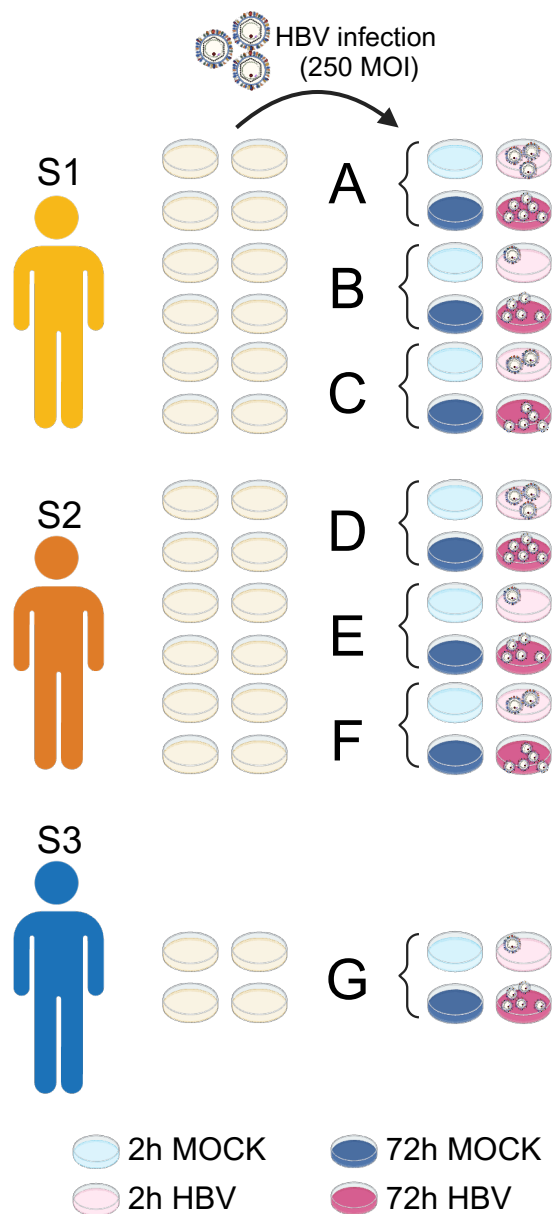

## ATAC sequencing and data analysis

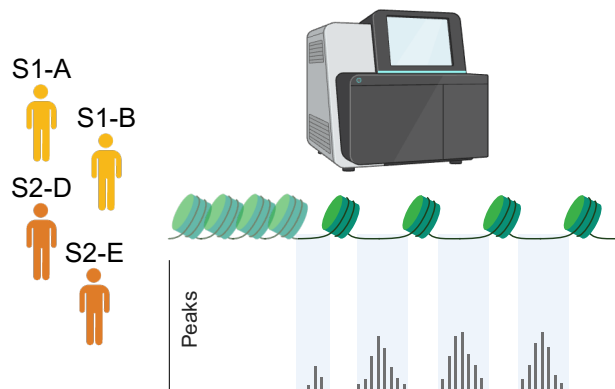

## RNA sequencing and data analysis

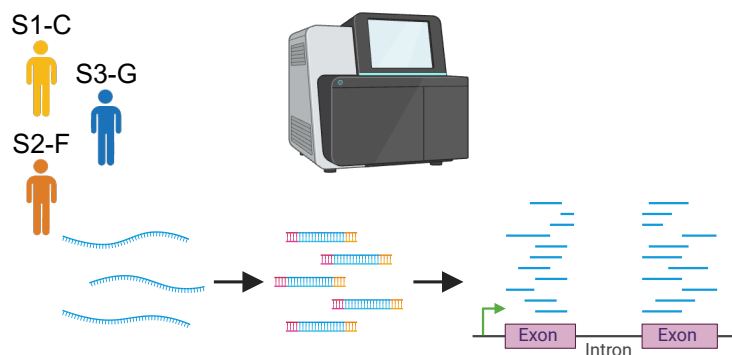

## DATA INTEGRATION

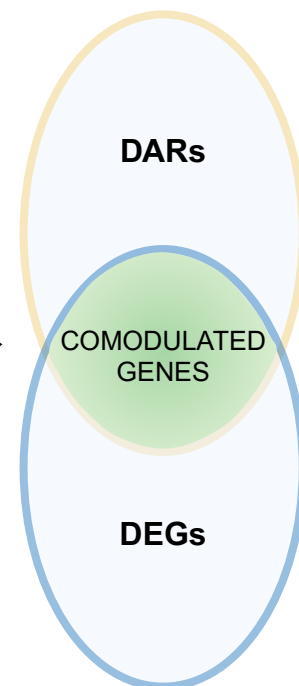

Supplement: Supplementary file 1 — Supplementary Material 1. [file 13046_2025_3414_MOESM1_ESM.pdf]

a

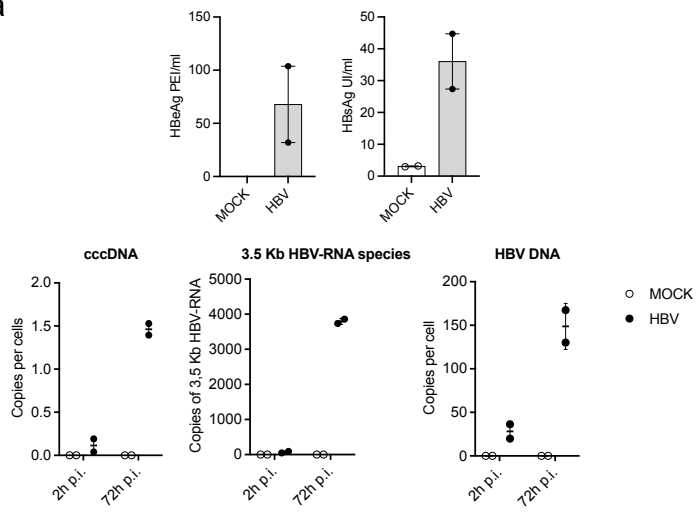

b

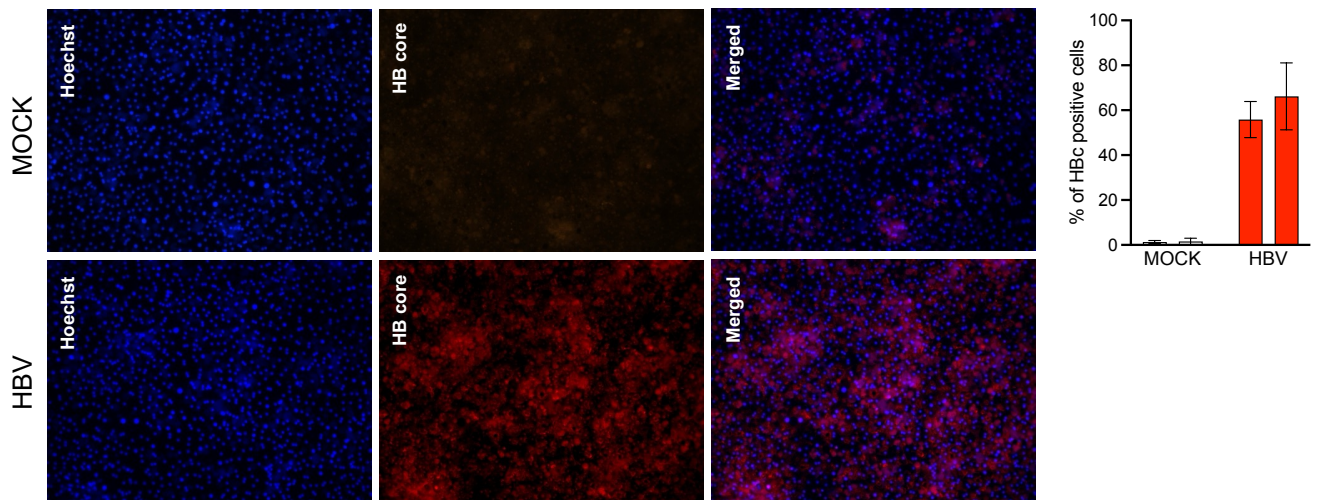

Supplement: Supplementary file 2 — Supplementary Material 2. [file 13046_2025_3414_MOESM2_ESM.pdf]

Figure S3

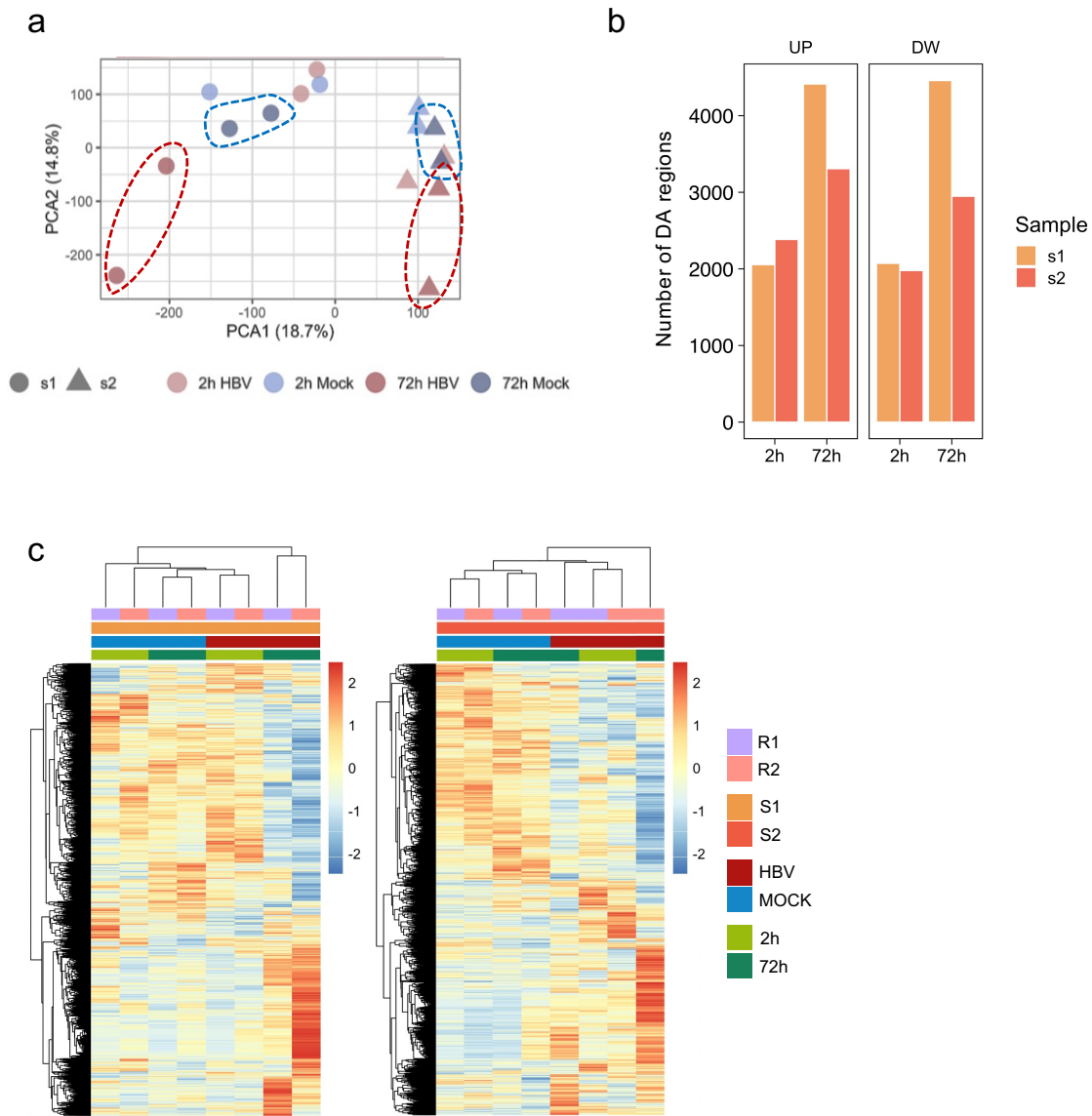

Supplement: Supplementary file 3 — Supplementary Material 3. [file 13046_2025_3414_MOESM3_ESM.pdf]

a

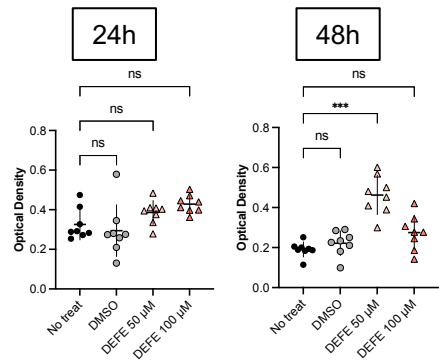

b

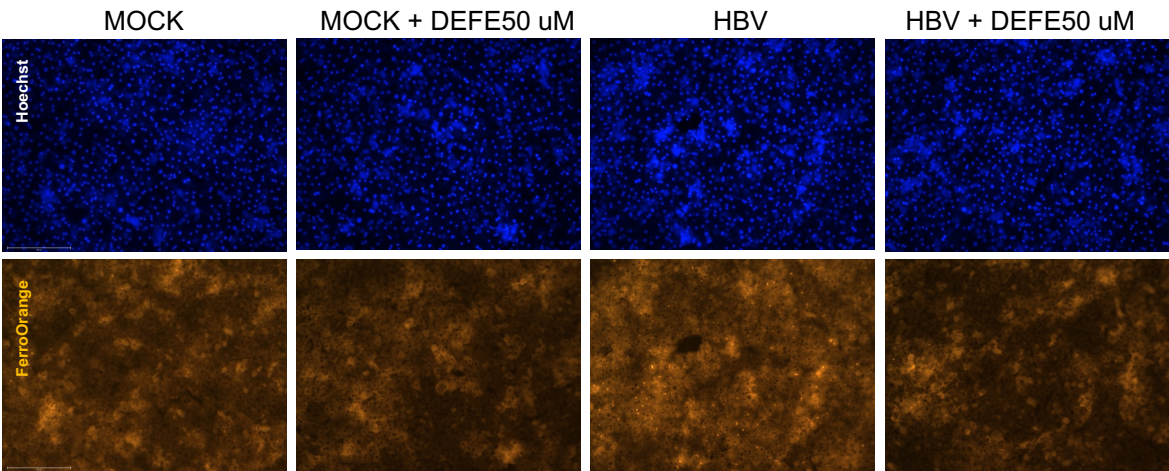

Supplement: Supplementary file 5 — Supplementary Material 5. [file 13046_2025_3414_MOESM5_ESM.pdf]

Figure S6

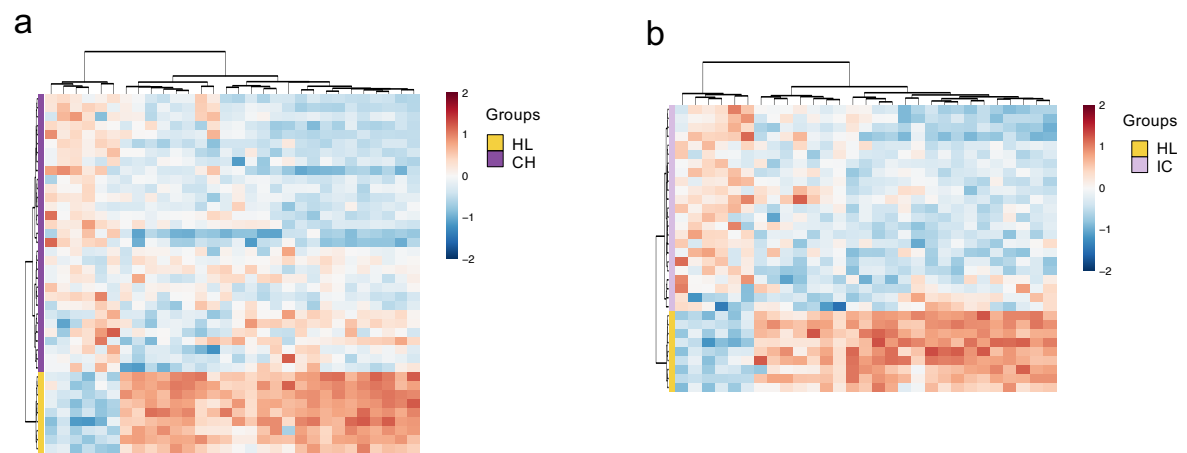

Supplement: Supplementary file 6 — Supplementary Material 6. [file 13046_2025_3414_MOESM6_ESM.pdf]

Figure S7

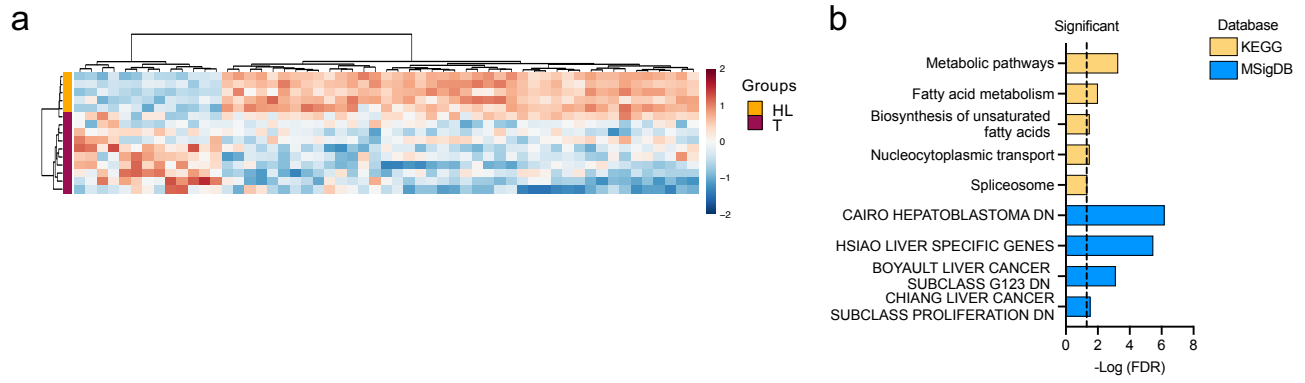

Supplement: Supplementary file 7 — Supplementary Material 7. [file 13046_2025_3414_MOESM7_ESM.pdf]

Figure S8

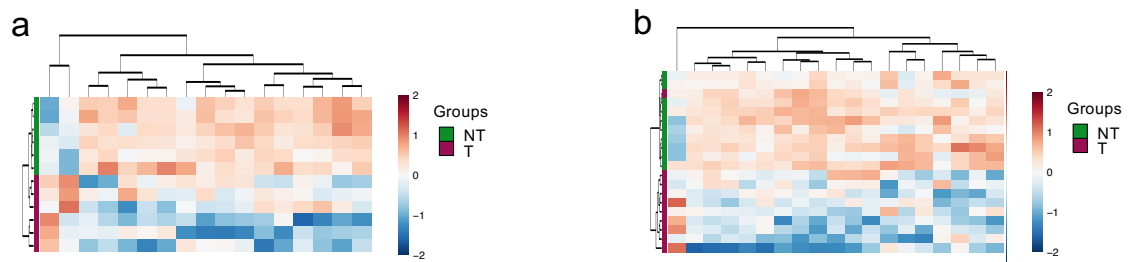

Supplement: Supplementary file 8 — Supplementary Material 8. [file 13046_2025_3414_MOESM8_ESM.pdf]

Figure S9

a

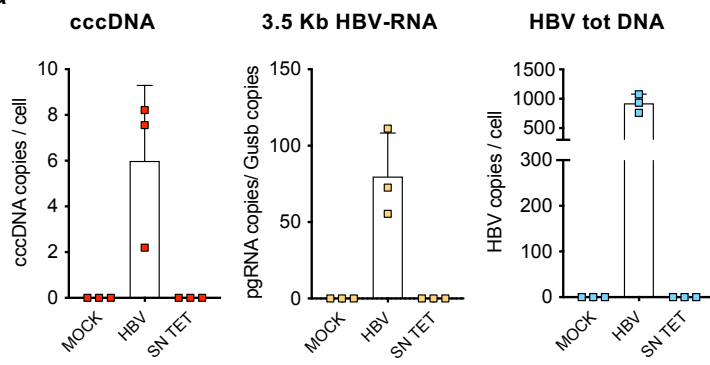

b

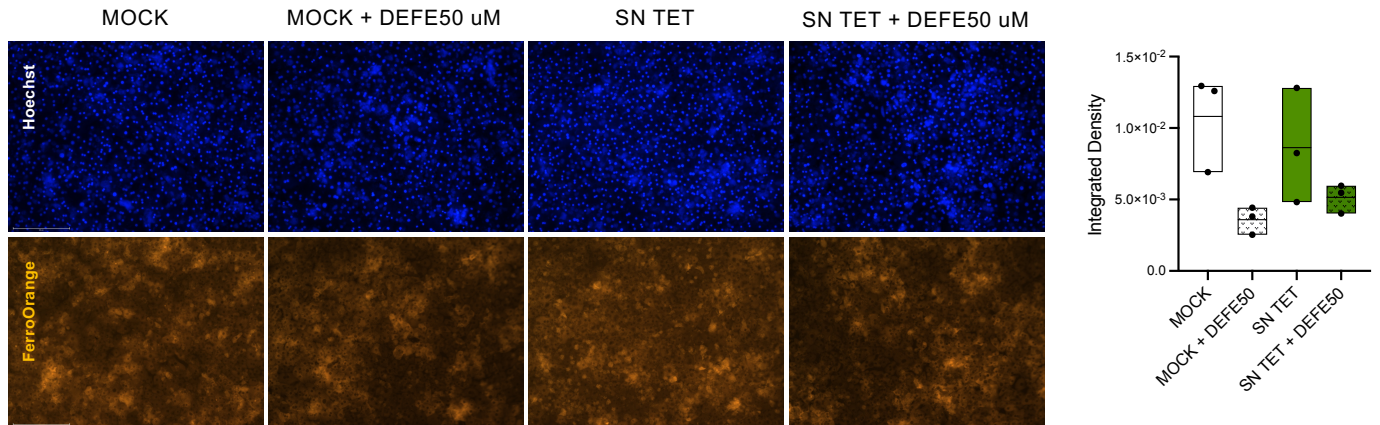

Supplement: Supplementary file 9 — Supplementary Material 9. [file 13046_2025_3414_MOESM9_ESM.pdf]
