# Supplementary material for: HBV-driven host chromatin accessibility changes affect liver metabolic pathways, iron homeostasis and promote a preneoplastic phenotype
Source: J Exp Clin Cancer Res. 2025 May 16;44:146. doi: 10.1186/s13046-025-03414-7 (PMC12082925; doi:10.1186/s13046-025-03414-7)
Supplement: Supplementary file 10 — Supplementary Material 10. [file 13046_2025_3414_MOESM10_ESM.docx]

***HBV-driven host chromatin accessibility changes affect liver metabolic pathways,***

***iron homeostasis and promote a preneoplastic phenotype***

Vincenzo Alfano^1^, Giuseppe Rubens Pascucci ^2,3^, Giacomo Corleone^4^, Massimiliano Cocca^1^, Francesca De Nicola^4^, Océane Floriot^1^, Alexia Paturel^1,5,^, Francesca Casuscelli Di Tocco^1^, Claude Caron de Fromentel^1^, Philippe Merle^1,6^, Michel Rivoire^7^, Massimo Levrero^1,6,#^, Francesca Guerrieri^1#^

^1^ IHU EVEREST - Institut of Hepatology Lyon, UMR UCLB1 INSERM U1350 PaThLiv, 69004 Lyon, France

^2^ Research Unit of Clinical Immunology and Vaccinology, Bambino Gesù Children's Hospital, 00165 Rome, Italy.

^3^ Center for Life Nano Science (CNLS), Istituto Italiano di Tecnologia (IIT), 00161 Rome, Italy

^4^ SAFU Laboratory, Department of Research, Advanced Diagnostics, and Technological Innovation, IRCCS Regina Elena National Cancer Institute, 00144 Rome, Italy

^5^ Université Catholique de Lyon (UCLy), 69002 Lyon, France

^6^ Department of Hepatology, Croix Rousse Hospital, Hospices Civils de Lyon, 69004, Lyon, France

^7^ INSERM U1052, Centre de lutte contre le cancer Léon Bérard (CLB), 69003 Lyon, France

**Table of contents:**

- Supplementary Materials and Methods

- Supplementary figures

- Supplementary References

***Supplementary Materials and Methods***

***Primary culture of human hepatocytes, HBV infection and pharmacological treatments.***

Primary Human Hepatocytes (PHHs) were isolated from normal liver tissue samples of HBV, HCV and HIV negative adult patients undergoing lobectomy or segmental liver resection for medically required purposes unrelated to this research program (Prof. M. Rivoire, Centre Leon Bérard, Lyon, France, ministerial agreements #AC-2013−1871 and DC-2013−1870). Signed informed written consents in compliance with the requirements of the local ethical committee were obtained from all patients before surgery. PHHs were prepared using the protocol described in Le Cluyse & Alexandre (1) with minor modifications. Liver samples are first perfused in Solution I (NaCl 58,44 M, KCl 74,56 M, Na2HPO4, 2H2O 177,99 M, Hepes 0,6%, EGTA 0,5 mM; pH7,4) and then in Solution II (NaCl 58,44 M, KCl 74,56 M, Na2HPO4, 2H2O 177,99 M, Hepes 0,6%, CaCl2 110 M) containing 0,4 mg/ml of collagenase from Clostridium histolyticum (Sigma-Aldrich, #C5138). Cells are seeded at 2,5x10^5 cells per cm² on collagen type IV (Corning, #354236) pretreated plates and cultured overnight in William’s medium (Life Technologies, #22551-089) supplemented with 10% Fetal Clone II (GE Healthcare), 1% penicillin/streptomycin (Invitrogen, #15140122), 1% Glutamax X100 (Invitrogen, #35050038), 5 µg/ml Insulin (Sigma–Aldrich, #I9278) and 5×10-7 M hydrocortisone (Upjohn Laboratories). PHH are then extensively washed in serum-free medium, kept in serum-free medium for 24 hours to counter select the growth of contaminating fibroblast and endothelial cells and then plated in complete William’s medium. PHHs were treated with 2% of DMSO (Sigma–Aldrich, #D2650) for 24 hours before infection and then incubated for 16 h with either an HBV inoculum produced in HepAD38 cells (see below) at a multiplicity of infection of 250 virus genome equivalents (vge) per cell, in presence of 2% of DMSO and 4% PEG-8000 (Polyethylene glycol, Sigma–Aldrich, #1546605) (HBV-infection) or with cell culture medium supplemented with 2% of DMSO 4% PEG-8000 (MOCK-infection). IFNα2a treatment (Roferon-A, Roche) on PHH was administered post infection (1000 UI/ml) and renewed every 48h until the desired time points (1000 UI/ml). Cells were treated with Deferasirox 50 μM and 100μM at 24h, 96h and 6 days post infection. To check whether other components present, beyond the infectious HBV viral particles, in the highly concentrated PEG precipitated supernatant from HBV-producing cells, might impact on the results we compared MOCK infection with the supernatant from HepAD38 cells grown in the presence of tetracycline (SN-TET), processed, and prepared in the same way as the infectious inoculum. As shown in the Figure S9a, cccDNA, 3.5 Kb HBV-RNAs and total HBV DNA could not be detected in PHHs exposed to SN-TET for 8 days. Moreover, there was no difference in iron uptake in MOCK-infected PHHs compared to PHHs exposed to SN-TET (Figure S9b). Finally, the basal iron uptake levels were similarly reduced in MOCK-infected PHHs and PHHs exposed to SN TET (Figure S9b).

***Patients and liver samples.*** RNA samples from matched tumour (T) and non tumour (NT, at least 2 cm distant from the T) tissues of 10 HBV-related HCC patients from 3 French clinical centres (Lyon Croix Rousse and Toulouse Hospitals and the French National Biological Resource Centres) have been retrospectively studied (Agreement DC-2008−235). RNAs from normal liver tissue samples of HBV, HCV and HIV negative adult patients undergoing lobectomy or segmental liver resection for medically required purposes unrelated to this research program at the Centre Leon Bérard (CLB) cancer center (ministerial agreements #AC-2013−1871 and DC-2013−1870) were used as controls. Signed informed written consents in compliance with the requirements of the local ethical committee were obtained from all patients before surgery.

***HBV virus preparation.*** The HBV inoculum used in this study was prepared from HepAD38 cells as previously described (2). HepAD38 is an HepG2-derived stable cell line Carrying a 1.3 HBV transgene (genotype D, serotype ayw) under the control of a *tet-off* promoter. HepAD38 cells were cultured in HYPER*Flask*^®^ (Corning, #10020) coated with type IV collagen (Corning, #354236) in 550 ml of complete DMEM-F12 medium supplemented with 10% decomplemented FBS (Gibco, #10270-106), 1% penicillin/streptomycin (Invitrogen, #15140122) and 1% sodium-pyruvate (Invitrogen, #11360039). Supernatants (550 ml/flask) were harvested twice a week for 3-4 months, clarified first through 0.45 μm and then 0.22 µm filters (Millipore, #10785534) and precipitated overnight at 4 °C with 8% PEG-8000 (Sigma–Aldrich, #1546605), The precipitates were centrifuged at 3500 g for 1 hour and the pellets resuspended in Opti-MEM (Invitrogen, #31985070) to achieve a 50-to-100-fold concentration. After DNA extraction (QIAmp Ultrasens Virus kit, Qiagen) the HBV inoculum was tittered by qPCR using serial dilutions of an HBV plasmid to build a standard curve. Primers are detailed in Table S1. All viral preparations were tested for the presence of endotoxins (Lonza Verviers, Belgium).

***Total DNA/RNA extraction and reverse transcription.*** Total cellular DNA was extracted using the MasterPure™ Complete DNA/RNA Purification Kit (Epicentre, #MC85200) according to manufacturer's recommendations. Total cellular RNA was extracted with the Extract-All reagent (Eurobio, #GEXEXT040U) following the manufacturer’s protocol. Extracted RNA was treated for 1 h at 37 °C with RNAse-free DNase I (Invitrogen, #AM2222), which was subsequently inactivated at 70 °C for 20 min. RNA reverse transcription was performed using the Superscript^®^ IV RT kit (Invitrogen, #18090010), according to manufacturer's instructions.

***Chromatin Tagmentation and Sequencing.*** 50 000 HBV or MOCK-infected cells harvested 2- and 72-hours post-infection were used for the transposase reaction, which was carried out essentially as described in Buenrostro et al. (3). PHHs were trypsinized (Trypsine 0,5% EDTA; Invitrogen, #25300054) at 37°C and the reaction was stopped by adding cell cultured medium on ice. After a wash in cold PBS 1X (Eurobio, #CS1PBS01KBP) cells were centrifugated at 500g for 5 minutes at 4°C, the pellets resuspend the in 50 µl of cold nuclei lysis buffer (10mM Tris-HCL [pH7,4], 10mM NaCl, 3mM MgCl2, 0,1% NP-40). After 30 minutes, the cells were centrifuged at 500g, for 5 minutes at 4°C, the supernatants removed, and pelleted nuclei used for the tagmentation protocol. Briefly, pellets were resuspended in the transposase reaction mix: 25 μL 2xTD buffer (Illumina Cat. FC-121-1030), 2.5 μL Tn5 transposase (Nextera Illumina) and 22.5 μL nuclease-free water) for 40 min at 37 °C and then purified on Qiagen MinElute columns. Libraries fragments were amplified using 1× NEBNext High fidelity 2x PCR masterMix (M0541) and 1,25 μM of custom Nextera PCR primers using the following PCR conditions: 72 °C for 5 min; 98 °C for 30 s; and thermocycling at 98 °C for 10 s, 63 °C for 30 s and 72 °C for 1 min. Library amplification were monitored by qPCR on 1/10 of the reaction after 5 cycles in order to avoid saturation and the risk of GC and size bias. Libraries were typically amplified for a total of 10–15 cycles followed by size-selection using AMPure beads and library quality control by Bioanalyzer (Agilent). Each ATAC-sequencing library was pair-end sequenced (150 cycles) to an average of 70 million reads per sample using a NextSeq V2 high output sequencing kit on NextSeq 500 Illumina platform.

***ATAC-seq data processing.*** The ATAC-sequencing reads quality was assessed with the FastQC v0.11.9 tool (http://www.bioinformatics.babraham.ac.uk/projects/fastqc/). Reads were aligned to the reference genome hg19 using bowtie v2.3.5.1 (4) with default parameters and processed downstream with SAMtools v1.7 (5). BAM files were deduplicated with GATK v4.1.9.0 (6) M*arkDuplicates* with default parameters, sorted and indexed with SAMtools v1.7 *sort* function.

Peaks were called with MACS2 v2.2.6 (7) (parameters: *--format AUTO --nomodel --shift -100 --extsize 200 -B --SPMR --call-summit -q 0.01 -g hs*). The bedGraphToBigWig v4 (8) tool was used to obtain BigWig files from BedGraph files. The *intersect* function of the bedtools suite v2.29.2 (9) was used to remove all peaks matching blacklisted regions (https:// www.encodeproject.org/files /ENCFF001TDO/@@download/ENCFF001TDO.bed.gz).

To evaluate the differential enrichment of ATAC-seq peaks, we first built a cumulative list (master list) of all the accessible regions identified in the profiling of each sample. Narrow peak files of each sample were concatenated, sorted, and merged with bedtools *merge* function to obtain the list of all accessible profiles. We then computed the enrichment of each peak in the master list by building a matrix of the peak reads counts of all samples using the bedtools *multicov* function, excluding duplicated reads. Peaks with mean counts higher than 5000 were considered outliers and discarded from the analysis. Reads counts were normalized using the edgeR (10) package’s function calcNormFactors by the Trimmed Mean of M values (TMM) (11) normalization and extracting the Counts Per Million (CPM) by the cpm function. Principal Component Analysis (PCA) was performed on normalized counts using the R prcomp function (https://www.R-project.org/) by scaling and centering the distributions. For each sample and for each time point, we filtered out peaks with a CPM mean lower than 10 in HBV or MOCK. Then we calculated a Ranking Index (RI) by dividing the CPM distributions into percentiles ranging from 0 (lowest enrichment) to 100 (highest enrichment). As the final output, we obtained a matrix of the RI assigned to the peaks of the master list in each sample. To select differential sites, we calculated the difference in RI between HBV and relative MOCK (delta = HBV RI - MOCK RI) and set a delta score threshold of +/- 20 to distinguish between background and real differences in a significant manner. To associate the nearest gene to each peak, we used Homer's annotatePeaks.pl function (v. 4.10) (12) with the default parameters. We further investigated the MOTIFs associated with differentially accessible sites using Homer's findMotifsGenome.pl function with "size given" parameter. Heatmap profiles were obtained respectively by merging the signal of all replicates of each group and then plotted using DeepToolsv.3.5.0 (13) functions computeMatrix (parameters: *scale-regions -a 2000 -b 2000 –region --skipZeros –missingDataAsZero -sortRegions descend*) and plotHeatmap.

***RNA sequencing and analysis.*** RNA was extracted with the RNAeasy mini kit (Qiagen, #74104) and treated with the RNAse Free DNAse set (Qiagen, #79254) and controlled for quality on a Bioanalyzer (Agilent). mRNA libraries were prepared using the TruSeq® Stranded RNA HT (w/Ribo-Zero™) kit to increases the depth of sequencing. Each library was pair-end sequenced with a range of 66 – 103 million reads across all samples using 75x2 cycles on a NextSeq 500 Illumina platform. The sequencing quality of each sample was assessed with FASTQC v.0.11.9. (http://www.bioinformatics.babraham.ac.uk/projects/fastqc/). RNA-seq reads were aligned against the Ensembl hg19 transcriptome and quantified using Kallisto v.0.46.2 with the -b 60 parameter (14) and Sleuth v.0.30.0 (15). Transcripts aligned on the non-canonical chromosome and transcripts with zero counts in all samples have been removed from the downstream analysis. PCA was performed on Transcripts Per Million (TPM) using the R prcomp function (https://www.R-project.org/) by scaling and centering the distributions. For each sample and for each time point, all transcripts with a TPM mean lower than 5 were filtered out. As done for ATAC-seq peaks, we assigned a Ranking Index (RI) based on TPM distribution to each transcript. Differentially Expressed Transcripts (DETs) were selected by calculating the differential RI between HBV and relative MOCK (delta = HBV's RI - MOCK's RI) and using +/- 20 as filtering threshold. This threshold was chosen as a balanced value to ensure discrimination between background noise and genuine differential activity. To get a unique list of Differentially Expressed Genes (DEGs) from the three samples (Fig. 3a), we calculated the mean delta values of the DETs for each gene. Then, we considered only genes with a mean delta value greater than 20 or lower than -20.

***Correlation analysis.*** To quantify the level of correlation between the already defined ATAC-seq DARs and RNA-seq DE genes at 72h p.i., first, we calculated the average CPM value for all DARs across all samples (union of DARs from S1 and S2). To obtain a single value in the case of multiple DAR tagging the same gene, we calculated the mean CPM value across all the selected regions. Subsequently, we computed the average log2 fold change between the HBV condition and the mock condition per gene. For the expression data, we selected only DETs in each sample and calculated the average TPM value for each gene. We then calculated the average log2 fold change by gene between the two study conditions across all samples. We intersected the two resulting gene lists, from ATAC and RNA dataset, and calculated the Spearman correlation between the Log2 fold changes (as a proxy of chromatin accessibility and gene expression differentiation).

***Ingenuity Pathway Analysis (IPA) and Gene Ontology (GO) analysis.*** To identify important pathways, we performed an enrichment analysis of genes that were commonly upregulated after 72 hours in S1, S2, and S3. For this analysis, we utilized the IPA database (16). Furthermore, we measured the enrichment of IPA Disease and Function terms by providing RNA-seq data of genes with matching chromatin accessibility profiles, which were then sorted by "Liver" and "Infection" associated terms. Gene Ontology, KEGG and Molecular Signature Database enrichment analysis were performed using the tool ShinyGO 0.76 on deregulated genes (17).

***Digital droplet PCR (ddPCR).*** A 22-μL reaction mixture was prepared comprising 11 μL of 2X ddPCR Supermix^TM^ for probes (no dUTP) (Bio-Rad), 1.1 μL of primers and probe mix, and 5 μL of cDNA or DNA. Nucleic acid inputs were adjusted to have acceptable negative events: 2.5ng for pgRNA duplex PCR, 50ng for cccDNA singleplex PCR and 10ng for HBV total DNA. The several PCR in duplex were done combining HBV (Pa03453406_s1, Thermofischer) with the human control HBB gene (#Hs00758889_s1, Thermofischer) for DNA, pgRNA with the human control GUSB gene (#Hs99999908_m1, Thermofischer) for cDNA and the singleplex cccDNA Droplet formation was carried out using a QX100 droplet generator. Subsequent amplification was performed in the C1000 Touch^TM^deep-well thermal cycler (Bio-Rad) with a ramp rate of 2 °C/s and the lid heated to 105 °C, according to the Bio-Rad recommendations. First, the enzyme was activated at 95 °C for 10 min followed by 40 cycles of denaturation at 94 °C for 30 s and 60 °C for one minute. The enzyme was deactivated at 98 °C for 10 min and the reaction was kept at 4 °C.

***Quantitative polymerase chain reaction (qPCR) and Plasmid-Safe DNase treatment.*** Quantitative real time PCR was performed by using PowerUp SYBR Green Master Mix (Thermo Fisher Scientific, #A25742) and Taqman following the manufacturer’s suggested protocol. For the quantification of viral parameters or target genes, 10 nanograms of cDNA or total cellular DNA were used. Total HBV DNA and RNA were amplified following cycling conditions: a denaturation step for 20 s at 95 °C; and then 40 cycles of 3 s at 95 °C, 30 s at 60 °C. HBV DNA or messenger RNA (mRNA) relative amount was normalized over the expression of housekeeping gene RPLP0 for DNA and GUSb for mRNA (primers are detailed in Table S1). Quantitative PCR for pgRNA was performed using TaqMan MasterMix and FAM-probes (Power Up Master Mix, Life Technologies, #A25742) following cycling conditions: a denaturation step for 20 s at 95 °C; and then 40 cycles of 3 s at 95 °C, 30 s at 60 °C. Relative amount was normalized over the expression of GUSb. Target gene expression was obtained by normalizing target quantity *per* housekeeping gene GUSb quantity, used as a control gene.

For cccDNA quantification, 500 ng of total DNA were digested for 6 h, at 37 °C, with a Plasmid-safe DNase (Epicentre, #E3150K) to get rid of contaminating rcDNA (relaxed circular HBV DNA) species and heat inactivated for 30 min at 80°C, before being used in qPCR with cccDNA and HBB primers and probes. The amplification was monitored with an ABI 7500 Fast qPCR instrument. Oligonucleotides and probes are listed in Table S1.

***Enzyme-linked immunoassay (ELISA) for secreted HBV antigens.*** HBeAg and HBsAg were quantified in cell culture in supernatants from HBV infected PHHs using commercially available ELISA kits (Autobio, China, #CL0313-2 and #CL0311-2), according to the manufacturer's protocol.

***HBc detection by Immunofluorescence.*** Cells were fixed with 4% formaldehyde (Sigma-Aldrich, #F8775) in PBS 1X (Eurobio, #CS1PBS01KBP), permeabilized with 0.1% Triton X-100 (Sigma-Aldrich, #T8787) in PBS 1X and then treated with 1,5% of BSA (Euromedex, #04-100-812-E) in PBS 1X. Cells were then incubated for 1 hour at room temperature with an anti-HBc mouse monoclonal antibody (DAKO B0586; 1:500 dilution). After washing, cells were incubated with a goat anti-mouse Alexa Fluor 555-conjugated secondary antibody (Cell Signaling, #4409; 1:1000 dilution). Nuclei were counterstained using DAPI (Cell Signaling, #4083; 1:5000 dilution).

***Curated list of Iron metabolism-related genes.*** We selected twenty-three iron metabolism-related gene sets from the Molecular Signatures Database v5.1 (MSigDB) (http://software. broadinstitute.org/gsea/index.jsp) (complete list in Supplementary information) using the keyword “iron” to extract a unique list of 524 iron metabolism-related genes. The selected twenty-three iron metabolism-related gene sets:

*GOMF_IRON_ION_TRANSMEMBRANE_TRANSPORTER_ACTIVITY; GOBP_IRON_COORDINATION_ENTITY_TRANSPORT; GOMF_4_IRON_4_SULFUR_CLUSTER_BINDING;*

*GOBP_HEME_TRANSPORT;*

*GOBP_INTRACELLULAR_IRON_ION_HOMEOSTASIS; GOBP_IRON_IMPORT_INTO_CELL;*

*GOBP_IRON_ION_TRANSPORT;*

*GOBP_IRON_ION_HOMEOSTASIS; GOBP_IRON_ION_IMPORT_ACROSS_PLASMA_MEMBRANE; GOBP_IRON_ION_TRANSMEMBRANE_TRANSPORT; GOMF_2_IRON_2_SULFUR_CLUSTER_BINDING;*

*GOMF_IRON_ION_BINDING;*

*GOBP_CELLULAR_RESPONSE_TO_IRON_ION;*

*GOMF_FERROUS_IRON_BINDING;*

*GOBP_SEQUESTERING_OF_IRON_ION;*

*GOMF_FERRIC_IRON_BINDING;*

*GOBP_RESPONSE_TO_IRON_ION; GOMF_FERROUS_IRON_TRANSMEMBRANE_TRANSPORTER_ACTIVITY; HALLMARK_HEME_METABOLISM;*

*GOBP_HEME_METABOLIC_PROCESS;*

*HEME_BIOSYNTHETIC_PROCESS; GOBP_REGULATION_OF_IRON_ION_TRANSPORT; REACTOME_IRON_UPTAKE_AND_TRANSPORT*.

***Measurement of Intracellular Fe^+2^ ions.*** The BioTracker^TM^ FerroOrange Live Cell Dye (#SCT210, Merck KGaA, Darmstadt, Germany) was used according to the manufacturer's protocol for the detection of intracellular labile iron ions (Fe^+2^). FerroOrange is a cell-permeant dye that exhibits fluorogenic signal when bound to Fe^+2^. The MOCK and HBV-infected cells were rinsed and incubated with 1 µM FerroOrange and nuclei-staining dye Hoechst (100 nM) at 37°C, 5% CO_2_ for 30 minutes. After washing with PBS, the cells were observed with an inverted fluorescence microscope (Leica Biosystems, Wetzlar, Germany). Fe^+2^ fluorescence intensity was quantified with ImageJ software (18).

***Measurement of cell viability.*** PHHs were plated at a density of 80,000 cells per well, cultured for 3 days and then treated with DMSO, DEFE 50 μM, and DEFE 100 μM for 24 and 48 hours. Cell viability was assessed using the the MTT reduction colorimetric assay. The absorbance of solubilized purple formazan, that reflects the number of viable metabolic active cells, was quantified at a wavelength of 570 nm using a microplate spectrophotometer (Thermo Scientific™ Multiskan™ GO).

***Additional statistical analysis, comparison with GTEx data and differential expression analysis on RNA-seq cohorts.***

All additional statistical analyses were performed using GraphPad Prism version 9.5.1 GraphPad Software, La Jolla California USA, (https://www.graphpad.com/scientific-software/prism/). The GTEx database (18) was queried to extract mean expression levels as TPMs for genes relevant to our study. The data used for the analyses described in this manuscript were obtained from the GTEx Portal on 10/04/23. Raw expression data for HBV-related liver cancer (LIHC) patients were obtained from the TCGA Research Network (https://www.cancer.gov/tcga) using the TCGAbiolinks R package (19). All differential expression analyses were performed via the Deseq2 R package (20). Survival analysis on the whole TCGA-LIHC dataset was performed using the Kaplan-Meier method implemented in the online tool Kaplan-Meier plotter (https://kmplot.com/analysis/index.php?p=home) (21)

***Data Deposition.*** Database ATAC-Seq: GSE240183 study at: https://www.ncbi.nlm.nih.gov/ geo/query/acc.cgi?acc=GSE240183; Database RNA-Seq: GSE239860 study at: https://www. ncbi.nlm.nih.gov/geo/query/acc.cgi?acc=GSE239860; Database HCC-HBV: GSE251942 study at: https://www.ncbi.nlm.nih.gov/geo/query/acc.cgi?acc=GSE251942.

SUPPLEMENTARY FIGURES

**Figure S1. Experimental design for ATAC-seq and RNA-seq profiling following HBV infection.**

Primary human hepatocytes from three donors (S1, S2, S3) were used to investigate the host response to HBV infection at chromatin accessibility and gene expression levels. All replicates were infected with HBV at a multiplicity of infection (MOI) of 250 and harvested at two time points: 2 hours (pink) and 72 hours (magenta) post-infection, alongside matched mock-infected controls (ligth blue for the 2 hours and dark blue for the 72 hours).

For ATAC-seq, the PHHs from two donors (S1 and S2) were plated in triplicates (A–C for S1; D–F for S2). Two replicates per donor (A, B for S1; D, E for S2) were subjected to ATAC-seq analysis. For RNA-seq, one replicate from each donors (C for S1 and F for S2), as well as one replicate from a third independent donor (S3-G), were used.

Differentially accessible regions (DARs) and differentially expressed genes (DEGs) were subsequently identified and integrated to define gene sets exhibiting coordinated modulation at both chromatin and transcriptional levels in response to HBV.

**Figure S2**

1. Virological parameters in HBV-infected PHHs. Infections were carried out at 250 MOI (Multiplicity of Infection). HBe antigen (PEI/ml) and HBsAg (UI/ml) quantification in cell culture supernatants by ELISA assays in MOCK and HBV-infected PHHs (upper panels). Digital PCR (ddPCR) quantification of nuclear HBV cccDNA and total HBV-DNA (lower left and right panels), results are expressed as copies per cell. ddPCR quantification of intracellular 3.5 Kb HBV-RNA species (lower middle panel) at 2 hours (2h) and 72 hours (72h) post infection (p.i.).
2. Left panel: Representative images showing the localization of HBc (hepatitis B core protein, red) and nuclear DNA stained with Hoechst (blue). The merged panel illustrates the colocalization of HBc with nuclei in the field of view. HBcAg immunostaining, was >70% at 72h p.i.. Right panel: Graph plot of HBV HBc staining levels at 72h p.i. from the same PHH infections used for the ATAC-seq and RNA-seq experiments. The graph plot represents the mean + SD of the percentage of HBc positive cells from 6 different microscope fields (~100 cells / field) from MOCK infected PHHs and 6 fields (~100 cells / field) from HBV infected PHHs. The range of infected cells in the different replicates is 46% to 70.2% for sample 1 (S1) (mean 55.8% median 54.3%) and 43.3 to 79.3% for sample 2 (S2) (mean 66.2, median 71.6%), respectively.

**Figure S3.**

1. Principal components analysis (PCA) of the ATAC-seq global chromatin accessibility profiles of all MOCK and HBV-infected PHH samples at 2h p.i. and 72h p.i. S1 = donor 1; S2 = donor 2.
2. Bar plot showing the numbers of upregulated (UP) and downregulated (DW) regions in HBV vs MOCK samples.
3. Heatmaps of CPMs z-scores for the Differentially Accessible (DA) peaks at 2h p.i. and 72h p.i. in S1 (*left panel*) and S2 (*right panel*). Z-scores value are represented with a color scale from blue (negative values) to red (positive values)

**Figure S4.**

1. ATAC-seq density profiles in MOCK- and HBV-infected PHHs in the 2 replicates from S1 and S2 donors at 72h p.i. Intensities are represented with a color scale from blue (more accessible chromatin) to red (less accessible chromatin).
2. Digital PCR quantification of HBV virogical parameters: covalently closed circular DNA (cccDNA), HBV DNA, HBV RNA and pre genomic RNA (pgRNA) from HBV-infected PHHs untreated (HBV) or IFNα2A-treated (interferon) at 2 hours (2h) and 72 hours (72h) post infection (p.i.).
3. Heatmap of RNA-seq expression data from three MOCK and IFN-alpha treated PHHs replicates. All PHH samples showed a strong IFN response with the activation of several IFN-stimulated genes (ISGs).
4. CPM distribution for the Differentially Accessible Regions (DARs) located in the hotspots identified in chromosomes 1, 17 and 19 in HBV-infected vs MOCK PHHs (list and details in Table 2). *Red dots* represent DARs located between -5Kb and +5Kb from the TSS and display a reduced chromatin accessibility after HBV infection. *Green dots* represent DARs located between -5Kb and +5Kb from the TSS with increased chromatin accessibility after HBV infection. *Light blue dots* represent DARs located outside -5Kb / +5Kb limits.

**Figure S5.**

1. Cell viability was assessed using the MTT formazan dye assay in PHHs treated with DMSO, DEFE 50 μM and DEFE 100 μM for 24 hours (left panel) and 48 hours (right panel). Untreated PHHs (No treat) served as an additional control. Data show the Optical Density (OD) from two independent experiments, each conducted in quadruplicate. Horizontal bars and vertical lines represent the mean ± standard deviation (SD) for each group. Statistical analysis included one-way ANOVA and nonparametric tests (Kruskal-Wallis test and Dunn’s multiple comparisons test). *** : p < 0.0001.
2. Representative images of iron uptake incrases after HBV infection. Nuclei (Hoechst) and free Fe+2 ions (FerrOrange) staining in MOCK, MOCK treated (DEFE50 uM), HBV-infected and HBV infected and treated (DEFE50 uM) PHHs at 8d p.i. Magnification 20x.

**Figure S6.**

1. Heatmap of the 30 iron signature genes that are differentially expressed (24 down- and 6 upregulated) in CH patients (HBe pos and HBe neg CH; purple) vs non-HBV infected healthy livers (HL, dark yellow). Normalized counts are represented with a color scale from blue (negative) to red (positive).
2. Heatmap of the 29 iron signature genes that are differentially expressed (23 downregulated; 6 upregulated) in the liver of HBV inactive carriers (IC; e.g., HBe neg chronic infection (CI); light purple) vs non-HBV infected healthy livers (HL, dark yellow). Normalized counts are represented as in a).

**Figure S7.**

1. Heatmap of the 55 ATAC-Seq/RNA-seq co-regulated genes that are differentially expressed (42 down- and 13 up-regulated) in HBV-related HCCs (T) tissues (n=10; purple) vs Healthy Liver (HL) samples (n=5; dark yellow). Normalized counts are represented with a color scale from blue (negative) to red (positive).
2. KEGG pathways (Release 86.1; yellow bars) and MSigDB modules significantly enriched from the 56 genes differentially expressed in T vs HL (ShinyGO 0.76 tool) and sorted by– Log (FDR) values. Fold enrichment ranks the relative number of genes included in each enriched pathway (see Table S15).

**Figure S8.**

1. Heatmap of the 17 iron signature genes (18 down- and 1 up-regulated) differentially expressed in the 6 paired T (purple) vs NT (green) liver tissues from the HBV-related HCCs of the TCGA LIHC database. Normalized counts are represented with a color scale from blue (negative) to red (positive).
2. Heatmap of the 19 iron signature genes (18 down- and 1 up-regulated) differentially expressed in the 10 paired T (purple) vs NT (green) liver tissues from the HBV-related HCCs of the Lyon HBV HCC cohort. Normalized counts are represented as in a).

**Figure S9.**

1. Digital PCR quantification of HBV virogical parameters: covalently closed circular DNA (cccDNA), HBV DNA, HBV RNA and pre genomic RNA (pgRNA) PHHs exposed to SN-TET for 8 days.
2. Left panel: Nuclei (Hoechst) and free Fe+2 ions (FerroOrange) staining in PHHs exposed to a supernatant from HepAD38 cells grown in the presence of tetracycline (SN-TET) and SN-TET treated with DEFE50 uM. Magnification 20x. Right panel: Integrated density of Fe+2 ions staining (n = 3 experiments) at 8 days p.i. for the different experimental conditions.

***Supplementary References***

1. Lecluyse EL, Alexandre E. Isolation and culture of primary hepatocytes from resected human liver tissue. Methods Mol Biol. 2010;640:57–82.

2. Ladner SK, Otto MJ, Barker CS, Zaifert K, Wang GH, Guo JT, et al. Inducible expression of human hepatitis B virus (HBV) in stably transfected hepatoblastoma cells: a novel system for screening potential inhibitors of HBV replication. Antimicrob Agents Chemother. 1997;41:1715–1720.

3. Buenrostro JD, Wu B, Chang HY, Greenleaf WJ. ATAC-seq: A Method for Assaying Chromatin Accessibility Genome-Wide. Curr Protoc Mol Biol. 2015;109:21.29.1-21.29.9.

4. Langmead B, Salzberg SL. Fast gapped-read alignment with Bowtie 2. Nat Methods. 2012;9:357–359.

5. Li H, Handsaker B, Wysoker A, Fennell T, Ruan J, Homer N, et al. The Sequence Alignment/Map format and SAMtools. Bioinformatics. 2009;25:2078–2079.

6. McKenna A, Hanna M, Banks E, Sivachenko A, Cibulskis K, Kernytsky A, et al. The Genome Analysis Toolkit: a MapReduce framework for analyzing next-generation DNA sequencing data. Genome Res. 2010;20:1297–1303.

7. Zhang Y, Liu T, Meyer CA, Eeckhoute J, Johnson DS, Bernstein BE, et al. Model-based analysis of ChIP-Seq (MACS). Genome Biol. 2008;9:R137.

8. Kent WJ, Zweig AS, Barber G, Hinrichs AS, Karolchik D. BigWig and BigBed: enabling browsing of large distributed datasets. Bioinformatics. 2010;26:2204–2207.

9. Quinlan AR, Hall IM. BEDTools: a flexible suite of utilities for comparing genomic features. Bioinformatics. 2010;26:841–842.

10. Robinson MD, McCarthy DJ, Smyth GK. edgeR: a Bioconductor package for differential expression analysis of digital gene expression data. Bioinformatics. 2010;26:139–140.

11. Robinson MD, Oshlack A. A scaling normalization method for differential expression analysis of RNA-seq data. Genome Biol. 2010;11:R25.

12. Heinz S, Benner C, Spann N, Bertolino E, Lin YC, Laslo P, et al. Simple combinations of lineage-determining transcription factors prime cis-regulatory elements required for macrophage and B cell identities. Mol Cell. 2010;38:576–589.

13. Ramírez F, Ryan DP, Grüning B, Bhardwaj V, Kilpert F, Richter AS, et al. deepTools2: a next generation web server for deep-sequencing data analysis. Nucleic Acids Res. 2016;44:W160-165.

14. Pimentel H, Bray NL, Puente S, Melsted P, Pachter L. Differential analysis of RNA-seq incorporating quantification uncertainty. Nat Methods. 2017;14:687–690.

15. Bray NL, Pimentel H, Melsted P, Pachter L. Near-optimal probabilistic RNA-seq quantification. Nat Biotechnol. 2016;34:525–527.

16. Krämer A, Green J, Pollard J, Tugendreich S. Causal analysis approaches in Ingenuity Pathway Analysis. Bioinformatics. 2014;30:523–530.

17. Ge SX, Jung D, Yao R. ShinyGO: a graphical gene-set enrichment tool for animals and plants. Bioinformatics. 2020;36:2628–2629.

18. GTEx Consortium. The GTEx Consortium atlas of genetic regulatory effects across human tissues. Science. 2020;369:1318–1330.

19. Mounir M, Lucchetta M, Silva TC, Olsen C, Bontempi G, Chen X, et al. New functionalities in the TCGAbiolinks package for the study and integration of cancer data from GDC and GTEx. PLOS Computational Biology. 2019;15:e1006701.

20. Love MI, Huber W, Anders S. Moderated estimation of fold change and dispersion for RNA-seq data with DESeq2. Genome Biology. 2014;15:550.

21. Menyhárt O, Nagy Á, Győrffy B. Determining consistent prognostic biomarkers of overall survival and vascular invasion in hepatocellular carcinoma. Royal Society Open Science. 2018;5:181006.
