# Supplementary material for: HBV-driven host chromatin accessibility changes affect liver metabolic pathways, iron homeostasis and promote a preneoplastic phenotype
Source: J Exp Clin Cancer Res. 2025 May 16;44:146. doi: 10.1186/s13046-025-03414-7 (PMC12082925; doi:10.1186/s13046-025-03414-7)
Supplement: Supplementary file 11 — Supplementary Material 11. [file 13046_2025_3414_MOESM11_ESM.pdf]

Table S1.
Primers and probes for RT-qPCR and ddPCR

Digital droplet PCR and qPCR probes	Probes	
**GUSb**	#Hs99999908_m1 (Thermofischer)	
**HBB**	#Hs00758889_s1 (Thermofischer)	
**HBV total**	Pa03453406_s1 (Thermofischer)	
**cccDNA**	CATGGAGACCACCGTGAACGCCC	
**pgRNA**	AGGCAGGTCCCCTAGAAGAAGAACTCC	
	Forward (5'-3')	Reverse (5'-3')
**pgRNA**	GGAGTGTGGATTCGCACTCCT	AGATTGAGATCTTCTGCGAC
**cccDNA**	CCGTGTGCACTTCGCTTCA	GCACAGCTTGGAGGCTTGA
**HBV total**	Pa03453406_s1 (Thermofischer)	
**qPCR primers**	Forward (5'-3')	Reverse (5'-3')
**GUSb**	#Hs99999908_m1 (Thermofischer)	
**RLPO**	CACCATTGAAATCCTGAGTGATGT	TGACCAGCCCAAAGGAGAAG
**TFRC**	TGGACAGCACAGACTTCACC	ACGCCAGACTTTGCTGAGTT
**TF**	GAGACCACCGAAGACTGCAT	ACAGGCACCAGACCACACTT
**SLC11A2**	ATCCGATTTGCAGTCTGGAG	AGATGCAGCCCAGTAACCAC
