# Supplementary material for: HBV-driven host chromatin accessibility changes affect liver metabolic pathways, iron homeostasis and promote a preneoplastic phenotype
Source: J Exp Clin Cancer Res. 2025 May 16;44:146. doi: 10.1186/s13046-025-03414-7 (PMC12082925; doi:10.1186/s13046-025-03414-7)
Supplement: Supplementary file 12 — Supplementary Material 12. [file 13046_2025_3414_MOESM12_ESM.pdf]

Table S2.
ATAC-seq QC statistics

Sample	Total reads (M)	Duplication rate (%)	Number of peaks
2h HBV s1 1	65,78435	9,56657	62680
2h HBV s1 2	70,978304	20,94106	31188
2h HBV s2 1	61,675304	3,267439	13571
2h HBV s2 2	62,771598	5,900416	9262
2h Mock s1 1	68,168008	9,659244	41051
2h Mock s1 2	64,201582	16,66609	36479
2h Mock s2 1	60,933276	4,703471	29795
2h Mock s2 2	60,97274	3,915609	36194
72h HBV s1 1	96,962918	23,50326	25732
72h HBV s1 2	91,5356	41,86722	5321
72h HBV s2 1	60,194394	4,03551	6789
72h HBV s2 2	57,550042	8,340987	1682
72h Mock s1 1	91,008138	13,982518	83291
72h Mock s1 2	86,64117	22,424748	42362
72h Mock s2 1	62,286906	4,401186	36706
72h Mock s2 2	61,786246	4,336348	15163
