# Supplementary material for: HBV-driven host chromatin accessibility changes affect liver metabolic pathways, iron homeostasis and promote a preneoplastic phenotype
Source: J Exp Clin Cancer Res. 2025 May 16;44:146. doi: 10.1186/s13046-025-03414-7 (PMC12082925; doi:10.1186/s13046-025-03414-7)
Supplement: Supplementary file 13 — Supplementary Material 13. [file 13046_2025_3414_MOESM13_ESM.pdf]

Table S3.
Genomic coordinates and detailed information of DARs defining three hotspots on chromosome 1,17 and 19.

ID	Strand	Distance to TSS	Associated gene	Avg TMM Mock	Avg TMM HBV
chr1:151031782-151032291	+	61	CDC42SE1	12,93	12,03
chr1:155079793-155080316	+	-20297	EFNA1	17,66	9,36
chr1:155101483-155101827	+	1303	EFNA1	18,73	11,58
chr1:157282863-157283525	+	-174811	ETV3	12,85	12,99
chr1:155225186-155225820	+	-229	FAM189B	15,41	9,39
chr1:149783504-149784211	+	70	HIST2H2BF	16,57	10,63
chr1:152186554-152187240	+	9775	HRNR	12,44	23,90
chr1:154392002-154392294	+	-13108	IL6R-AS1	15,03	7,80
chr1:153714864-153715507	+	14660	INTS3	22,35	13,72
chr1:155145220-155146017	+	185	KRTCAP2	15,78	21,11
chr1:146951583-146952051	+	37882	LINC00624	14,17	8,33
chr1:156084364-156084690	+	25	LMNA	14,25	10,02
chr1:143686878-143687408	+	16	LOC100132057	15,28	9,13
chr1:147874390-147874876	+	-3	LOC100132057	16,18	8,56
chr1:160208206-160208598	+	-23137	LOC100287049	15,64	6,96
chr1:143243452-143243712	+	-41343	LOC102723769	8,44	34,89
chr1:143245651-143245885	+	-43529	LOC102723769	10,18	56,86
chr1:143344710-143345255	+	-142744	LOC102723769	15,89	49,73
chr1:150158389-150159289	+	25913	LOC105371433	24,16	13,60
chr1:150980672-150981037	+	14	MINDY1	13,31	6,38
chr1:151542200-151542921	+	24289	MIR554	11,58	11,83
chr1:150266036-150266593	+	53	MRPS21	14,68	7,83
chr1:149910943-149911491	+	-2445	MTMR11	24,07	15,10
chr1:146069770-146070321	+	-3790	NBPF11	18,20	11,00
chr1:149024649-149025173	+	84814	NBPF25P	4,71	13,79
chr1:144963031-144963531	+	-30916	PDE4DIP	24,39	13,32
chr1:151170724-151171332	+	0	PIP5K1A	14,63	13,27
chr1:156182588-156183396	+	213	PMF1-BGLAP	30,32	17,45
chr1:151371724-151372155	+	-109	PSMB4	14,71	8,67
chr1:151237766-151238284	+	10815	PSMD4	14,91	12,79
chr1:145507319-145508005	+	105	RBM8A	25,07	15,89
chr1:149513574-149514293	+	322	RNVU1-19	17,49	10,23
chr1:144491290-144492130	+	-105	RNVU1-4	9,98	13,77
chr1:151964813-151965367	+	1245	S100A10	16,24	9,68
chr1:153630934-153631575	+	76	SNAPIN	20,34	12,59
chr17:79936852-79937247	+	1545	ASPSCR1	15,07	9,77
chr17:79011853-79012363	+	3157	BAIAP2	12,27	11,89
chr17:77751484-77752113	+	-148	CBX2	9,11	14,48
chr17:71288180-71288545	+	19777	CDC42EP4	13,60	8,97
chr17:73992877-73993295	+	-3901	CDK3	17,68	5,26
chr17:77704641-77705094	+	163	ENPP7	13,75	11,73
chr17:80696068-80696531	+	2809	FN3K	22,84	13,93
chr17:80674358-80674895	+	45	FN3KRP	14,73	15,59
chr17:72369992-72370440	+	6571	GPR142	13,79	9,28
chr17:79300779-79301279	+	-17981	LINC00482	15,53	10,30

chr17:71791824-71792212	+	9057	LINC02092	15,53	8,78
chr17:70088814-70089333	+	20600	LINC02097	13,20	7,35
chr17:71706152-71706638	+	-27598	LOC100134391	13,09	6,71
chr17:73266345-73267039	+	111	MIF4GD	16,18	13,54
chr17:79868115-79868639	+	674	PCYT2	12,16	11,98
chr17:72189010-72189499	+	-10562	RPL38	20,08	11,83
chr17:72199552-72199965	+	-58	RPL38	11,85	10,44
chr17:73663140-73663742	+	23	SAP30BP	14,37	8,46
chr17:75283625-75284106	+	-107	SEPTIN9	14,63	12,28
chr17:75312067-75312740	+	-3193	SEPTIN9	20,62	13,06
chr17:75426205-75426759	+	-20349	SEPTIN9	26,50	15,09
chr17:74380275-74380859	+	-123	SPHK1	12,65	13,41
chr17:73178561-73179425	+	61	SUMO2	21,88	12,91
chr17:77967199-77967780	+	42181	TBC1D16	14,04	12,71
chr17:78003078-78003555	+	6354	TBC1D16	16,03	10,03
chr17:80543995-80544465	+	62090	WDR45B	16,95	29,69
chr19:1876061-1876440	+	9244	ABHD17A	11,58	11,02
chr19:6199399-6200050	+	64047	ACSBG2	13,22	14,36
chr19:8426802-8427274	+	-2001	ANGPTL4	25,20	13,86
chr19:6720518-6720981	+	-89	C3	24,28	13,64
chr19:13858337-13858916	+	-82	CCDC130	28,88	17,77
chr19:1260882-1261114	+	-8269	CIRBP	11,73	10,70
chr19:1266211-1266790	+	-2766	CIRBP	19,57	16,88
chr19:11376415-11376824	+	-3420	DOCK6	14,29	8,57
chr19:3500361-3501106	+	-62	DOHH	17,96	17,90
chr19:3983993-3984340	+	1294	EEF2	15,93	9,47
chr19:1284442-1284956	+	-1173	EFNA2	8,86	14,08
chr19:2475882-2476257	+	-55	GADD45B	15,19	15,67
chr19:2540694-2541173	+	64809	GADD45B	28,26	17,17
chr19:7197080-7197547	+	97111	INSR	13,73	10,66
chr19:7224189-7224601	+	70030	INSR	14,56	13,45
chr19:8399800-8400439	+	8026	KANK3	10,15	13,46
chr19:2456766-2457198	+	-25	LMNB2	16,11	9,51
chr19:4539946-4540443	+	-147	LRG1	13,54	12,23
chr19:4123766-4124482	+	2	MAP2K2	13,56	17,99
chr19:2634540-2634936	+	4025	MIR7850	16,54	7,58
chr19:6273724-6274182	+	6033	MLLT1	17,81	10,87
chr19:3346703-3347214	+	-12669	NFIC	13,48	11,90
chr19:797733-798036	+	433	PTBP1	14,02	11,81
chr19:10788418-10788939	+	-23436	QTRT1	21,29	13,51
chr19:660840-661261	+	2163	RNF126	15,04	14,14
chr19:6767376-6767845	+	-165	SH2D3A	12,54	13,36
chr19:4284213-4284852	+	5935	SHD	14,24	12,16
chr19:4457658-4458156	+	-116	UBXN6	12,46	12,96
chr19:4909877-4910377	+	-29	UHRF1	9,14	14,16
chr19:12099256-12099988	+	1190	ZNF433-AS1	13,93	7,47
chr19:7145492-7145961	+	76013	ZNF557	15,66	9,14
chr19:12441986-12442380	+	2264	ZNF563	14,19	5,26
chr19:11707957-11708815	+	88	ZNF627	26,81	12,99
