# Supplementary material for: HBV-driven host chromatin accessibility changes affect liver metabolic pathways, iron homeostasis and promote a preneoplastic phenotype
Source: J Exp Clin Cancer Res. 2025 May 16;44:146. doi: 10.1186/s13046-025-03414-7 (PMC12082925; doi:10.1186/s13046-025-03414-7)
Supplement: Supplementary file 20 — Supplementary Material 20. [file 13046_2025_3414_MOESM20_ESM.pdf]

Table S10.
List of 39 iron metabolism comodulated genes

Symbol	Ensembl Gene ID	Description
SDHB	ENSG00000117118	succinate dehydrogenase complex iron sulfur subunit B
EPB41	ENSG00000159023	erythrocyte membrane protein band 4.1
SELENBP1	ENSG00000143416	selenium binding protein 1
NDUFS2	ENSG00000158864	NADH:ubiquinone oxidoreductase core subunit S2
HAAO	ENSG00000162882	3-hydroxyanthranilate 3,4-dioxygenase
EPAS1	ENSG00000116016	endothelial PAS domain protein 1
RBM5	ENSG00000003756	RNA binding motif protein 5
ALDH1L1	ENSG00000144908	aldehyde dehydrogenase 1 family member L1
TF	ENSG00000091513	transferrin
CP	ENSG00000047457	ceruloplasmin
TFRC	ENSG00000072274	transferrin receptor
CAST	ENSG00000153113	calpastatin
SFXN1	ENSG00000164466	sideroflexin 1
GCLC	ENSG00000001084	glutamate-cysteine ligase catalytic subunit
EIF2AK1	ENSG00000086232	eukaryotic translation initiation factor 2 alpha kinase 1
CYP3A5	ENSG00000106258	cytochrome P450 family 3 subfamily A member 5
TFR2	ENSG00000106327	transferrin receptor 2
CTSB	ENSG00000164733	Cathepsin B
RNF19A	ENSG00000034677	ring finger protein 19A, RBR E3 ubiquitin protein ligase
ALAD	ENSG00000148218	aminolevulinate dehydratase
NCOA4	ENSG00000266412	nuclear receptor coactivator 4
CYP2C8	ENSG00000138115	cytochrome P450 family 2 subfamily C member 8
CAT	ENSG00000121691	catalase
PC	ENSG00000173599	pyruvate carboxylase
SLC11A2	ENSG00000110911	solute carrier family 11 member 2
PAH	ENSG00000171759	phenylalanine hydroxylase
ALKBH2	ENSG00000189046	alkB homolog 2, alpha-ketoglutarate dependent dioxygenas
MARK3	ENSG00000075413	microtubule affinity regulating kinase 3
HAGH	ENSG00000063854	hydroxyacylglutathione hydrolase
DPH1	ENSG00000108963	diphthamide biosynthesis 1
SLC46A1	ENSG00000076351	solute carrier family 46 member 1
VEZF1	ENSG00000136451	vascular endothelial zinc finger 1
SMAD4	ENSG00000141646	SMAD family member 4
NDUFS7	ENSG00000115286	NADH:ubiquinone oxidoreductase core subunit S7
C3	ENSG00000125730	complement C3
CYP4F12	ENSG00000186204	cytochrome P450 family 4 subfamily F member 12
CYP4F2	ENSG00000186115	cytochrome P450 family 4 subfamily F member 2
FBXO7	ENSG00000100225	F-box protein 7
TNRC6B	ENSG00000100354	trinucleotide repeat containing adaptor 6B
