# Supplementary material for: HBV-driven host chromatin accessibility changes affect liver metabolic pathways, iron homeostasis and promote a preneoplastic phenotype
Source: J Exp Clin Cancer Res. 2025 May 16;44:146. doi: 10.1186/s13046-025-03414-7 (PMC12082925; doi:10.1186/s13046-025-03414-7)
Supplement: Supplementary file 4 — Supplementary Material 4. [file 13046_2025_3414_MOESM4_ESM.pdf]

Figure S4

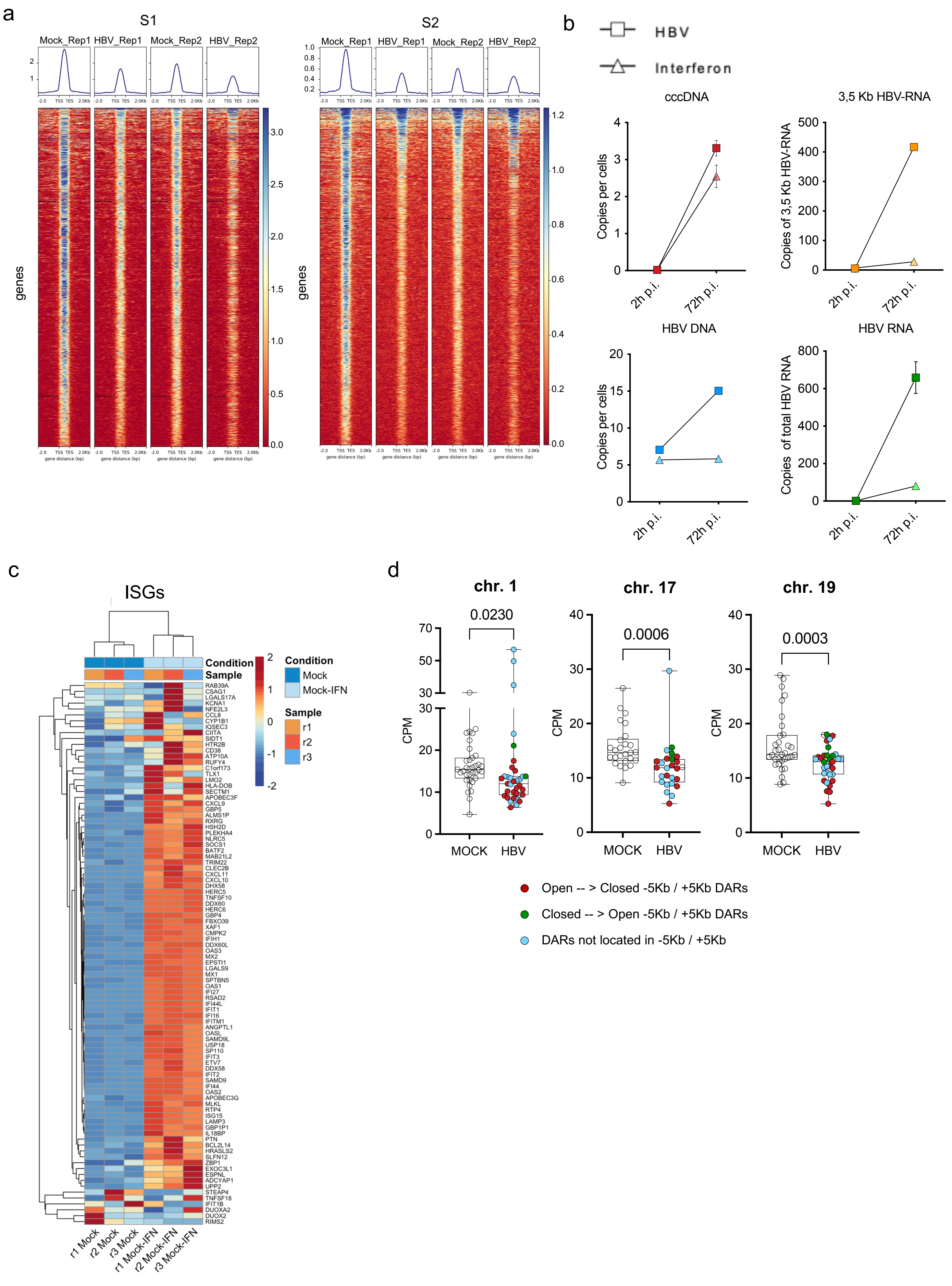

a
S1
Mock_Rep1
HBV_Rep1
Mock_Rep2
HBV_Rep2
S2
genes
gene distance (bp)
b
HBV
Interferon
cccDNA
3,5 Kb HBV-RNA
HBV DNA
HBV RNA
Copies per cells
Copies of 3,5 Kb HBV-RNA
Copies of total HBV RNA
2h p.i.
72h p.i.
c
ISGs
Condition
Sample
Mock
Mock-IFN
r1
r2
r3
r1 Mock
r2 Mock
r3 Mock
r1 Mock-IFN
r2 Mock-IFN
r3 Mock-IFN
d
chr. 1
chr. 17
chr. 19
0.0230
0.0006
0.0003
CPM
MOCK
HBV
Open -- > Closed -5Kb / +5Kb DARs
Closed -- > Open -5Kb / +5Kb DARs
DARs not located in -5Kb / +5Kb
